# Homogeneity of Supported Single‐Atom Active Sites Boosting the Selective Catalytic Transformations

**DOI:** 10.1002/advs.202201520

**Published:** 2022-07-09

**Authors:** Yujie Shi, Yuwei Zhou, Yang Lou, Zupeng Chen, Haifeng Xiong, Yongfa Zhu

**Affiliations:** ^1^ Key Laboratory of Synthetic and Biological Colloids Ministry of Education School of Chemical and Material Engineering Jiangnan University Wuxi Jiangsu 214122 P. R. China; ^2^ International Joint Research Center for Photoresponsive Molecules and Materials Jiangnan University Wuxi Jiangsu 214122 P. R. China; ^3^ College of Chemical Engineering Nanjing Forestry University Nanjing 210037 P. R. China; ^4^ College of Chemistry and Chemical Engineering Xiamen University Xiamen 361005 P. R. China; ^5^ Department of Chemistry Tsinghua University Beijing 100084 P. R. China

**Keywords:** active site, chemical transformations, homogeneity, selectivity, single‐atom catalyst

## Abstract

Selective conversion of specific functional groups to desired products is highly important but still challenging in industrial catalytic processes. The adsorption state of surface species is the key factor in modulating the conversion of functional groups, which is correspondingly determined by the uniformity of active sites. However, the non‐identical number of metal atoms, geometric shape, and morphology of conventional nanometer‐sized metal particles/clusters normally lead to the non‐uniform active sites with diverse geometric configurations and local coordination environments, which causes the distinct adsorption states of surface species. Hence, it is highly desired to modulate the homogeneity of the active sites so that the catalytic transformations can be better confined to the desired direction. In this review, the construction strategies and characterization techniques of the uniform active sites that are atomically dispersed on various supports are examined. In particular, their unique behavior in boosting the catalytic performance in various chemical transformations is discussed, including selective hydrogenation, selective oxidation, Suzuki coupling, and other catalytic reactions. In addition, the dynamic evolution of the active sites under reaction conditions and the industrial utilization of the single‐atom catalysts are highlighted. Finally, the current challenges and frontiers are identified, and the perspectives on this flourishing field is provided.

## Graphical abstract

It is highly desired to unravel the catalytic role of the homogeneity of active sites and develop appropriate strategies to modulate the homogeneity for better directing the selective chemical transformation. In this review, the authors highlight the homogeneity of active sites boosting the selective chemical transformations (selective hydrogenation/oxidation, Suzuki coupling, hydroformylation of olefins, etc.) and bridging homogeneous and heterogeneous catalysis.

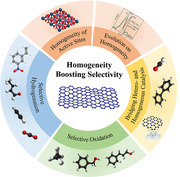

## 1 Introduction

Heterogeneous catalysts play a significant role in selective chemical transformations to reduce the occurrence of side reactions and drive the reactions toward the desired direction to produce more target products.^[^ ^1^, ^2^, ^3^ ^]^ With the advantages of being recyclable, environmentally friendly, and easy to separate from products compared to homogeneous catalysts, heterogeneous catalysts have been extensively explored and applied in academic research and industrial applications in the past decades.^[^ ^4^ ^]^ Supported metal catalysts where metal particles finely dispersed on the supports with high specific surface area play an important part in the industrially important catalytic reactions.^[^ ^5^, ^6^ ^]^ However, it is still very challenging to precisely design highly efficient supported metal catalysts for promoting selective transformations.

It has been generally believed that the adsorption strength and configuration of the reactants/intermediates on catalyst surface, to a large degree, modulate the selectivity, which depends on the composition, structure, and electronic state of active sites.^[^ ^7^, ^8^ ^]^ For example, *α*, *β*‐unsaturated aldehydes have various adsorption modes on the Pt surface according to different configurations of Pt species. On an extensive Pt (111) surface, both C=C and C=O bonds can be adsorbed via di‐*σ* mode. As C=C and C=O bonds are conjugated in one molecule, they can be co‐adsorbed via the 1,4‐di‐*σ* modes. On isolated Pt atoms, both C=C and C=O bonds can be adsorbed via *π* mode. Moreover, the C=O bonds can be adsorbed on Pt single atoms via the end‐on mode. The adsorption patterns of the C=C bonds all lead to the saturation of C=C groups, while the C=O di‐*σ* mode is inclined to the side reaction of decarbonylation. The absorption strength of C=O *π* mode is weak. The end‐on mode can promote the preferential hydrogenation of the C=O groups and the desired products will be obtained.^[^ ^9^ ^]^ One can conclude that the isolation of active sites of metal species provides effective methods for improving the selectivity of unsaturated alcohol.

In conventionally supported metal nanoparticles (NPs)/clusters, the interfacial atoms located at the boundary of metal species and support are generally considered as the active sites. It has also been reported that the electronic structure and adsorption properties of those interfacial atoms are distinctive from those in other locations.^[^ ^10^, ^11^ ^]^ However, due to non‐identical metal atoms, geometric shape, and morphology, supported metal NPs/clusters usually have non‐uniform active sites with diverse geometric configurations and local coordination environments, which leads to various adsorption states of surface species. As a result, side reactions are often inevitable when non‐uniform NPs/clusters are used in selective chemical transformations.

When metal species are downsized to the atomic level, the single‐atom catalysts (SACs) are formed, where all the isolated metal species are directly in contact with the surface sites, thus maximizing the number of interfacial atoms. Whereas in NPs, the unexposed inner atoms cannot be in contact with the support to form interfacial sites.^[^ ^12^ ^]^ For the uniform active sites, SACs tend to perform better in selective transformations than their NP counterparts.^[^ ^13^ ^]^ Taking Au_1_/CeO_2_ as an example, the Au SACs show much higher selectivity than that of Au/CeO_2_ NP catalyst for selective oxidation of benzyl alcohol to benzaldehyde.^[^ ^14^ ^]^ It's noteworthy that the lattice oxygen atoms play a critical role in determining the high selectivity, serving as an oxidizing agent. The isotope experiment confirms that singly dispersed SACs can activate lattice oxygen sites, which participate in the oxidation process and are more selective than molecular oxygen activated on the metal surface for the restricted geometry of the active sites in SACs. Since the heats of adsorption for benzyl alcohol on SACs and NP catalysts are both low, the great catalytic performance is mainly determined by the weak adsorption of benzaldehyde as facile desorption reduces overoxidation. Therefore, besides lattice oxygen sites, the strength of aldehyde adsorption influences selectivity as well.

The SACs, as an important bridge between heterogeneous and homogeneous catalysts, exhibit great potential for enabling rational use of metal resources and maximizing atom utilization efficiency in numerous selective chemical transformations.^[^ ^15^, ^16^, ^17^, ^18^ ^]^ However, it will be a long time before extensive industrial applications since some challenges still need to be settled presently. On the one hand, scientists have been investigating the strategies for the large‐scale synthesis of SACs with high loading of metal species. On the other hand, the problem of how to stabilize the SACs during reaction processes deserves further research.

In this review, we first attempt to overview the fabrication and characterization of uniform active sites on various catalyst systems. Then the catalytic applications of uniformity of active sites boosting various chemical transformations, including selective hydrogenation reactions, selective oxidation reactions, Suzuki coupling reactions, and other catalytic reactions, will be particularly discussed. What's more, the dynamic evolution of uniformity of active sites under reaction conditions will be discussed. Last but not least, challenges and opportunities on the study and practical application of homogeneity of active sites in the community of catalysis will be prospected.

## 2 Supported Single‐Atom Catalysts (SACs)

It was reported that small particles (2–5 nm) of nano gold exhibited high activity in CO oxidation, selective oxidation, and other reactions in 1987.^[^ ^19^, ^20^ ^]^ During the same period, Bruce Gates first proposed the concept of isolated mononuclear when catalyzing alkane hydrogenolysis (C‐C bond breaking) on metal surfaces, which exhibits excellent selectivity.^[^ ^21^ ^]^ After that, the concept of atomic dispersion proved by the extended X‐ray absorption fine structure (EXAFS) data was proposed by Iwasawa and co‐workers and they also found the atomically dispersed Pt species in the catalysts were as active as Pt particles for the reaction in 1999.^[^ ^22^ ^]^ The concept of atomic dispersion is of great significance, which is still used to describe SACs even today. Thomas and co‐workers introduced the concept of single‐site heterogeneous catalysts in 2005.^[^ ^23^ ^]^ The “single‐site” may consist of one or more atoms, as long as the supported species show the same composition, structure, and properties. And in 2007, the high‐definition spots of single‐site catalysts were captured by high‐angle annular dark‐field scanning transmission electron microscopy (HAADF‐STEM) for the first time, which provided a characterization strategy for subsequent research on atomically dispersed catalysts.^[^ ^24^ ^]^

**Figure 1:**
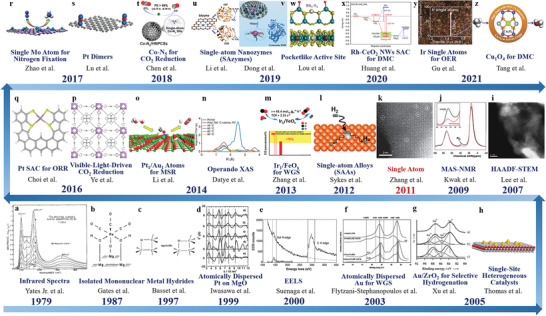
The journeys of isolated metal catalysts. a) Infrared spectra for ^12^CO adsorbed on Rh for increasing CO coverage. T = 295K. Reproduced with permission.^[^ ^27^ ^]^ Copyright 1979, AIP Publishing. b) Structure of the MgO‐supported rhenium subcarbonyl, a catalyst precursor. Reproduced with permission.^[^ ^21^ ^]^ Copyright 1987, Springer Nature. c) A metallocycle proposed as the transition state in alkane metathesis reaction. Reproduced with permission.^[^ ^28^ ^]^ Copyright 1997, AAAS. d) k^3^‐weighted EXAFS oscillations (k^3^ *χ*(k)) for PtMo_6_/MgO a), Pt(A)/MgO b), Pt/MgO prepared from PtCl_2_ c), Ni/MgO d) and Fe/MgO e). Reproduced with permission.^[^ ^22^ ^]^ Copyright 1999, Elsevier. e) EELS spectrum showing the Gd *N* edge and C *K* edge. Reproduced with permission.^[^ ^29^ ^]^ Copyright 2000, AAAS. f) XPS spectra of Au/CeO_2_ for water gas shift (WGS) reaction. Reproduced with permission.^[^ ^30^ ^]^ Copyright 2003, AAAS. g) XPS spectra (Au 4f) of 0.76 % Au/ZrO_2_ catalysts calcined at different temperatures: (a) 500, (b) 300, and (c) 200  °C. Reproduced with permission.^[^ ^31^ ^]^ Copyright 2005, Wiley‐VCH. h) Single‐site heterogeneous catalysts. Reproduced with permission.^[^ ^32^ ^]^ Copyright 2013, American Chemical Society. i) HAADF‐STEM image of 0.03 wt.% Pd/meso‐Al_2_O_3_. Reproduced with permission.^[^ ^24^ ^]^ Copyright 2007, Wiley‐VCH. j) The ^27^Al MAS‐NMR spectra of *γ*‐Al_2_O_3_ (black) and 10 wt.% Pt/*γ*‐Al_2_O_3_ (red). Reproduced with permission.^[^ ^33^ ^]^ Copyright 2009, AAAS. k) HAADF‐STEM image of the Pt_1_/FeO_x_ SAC. Reproduced with permission.^[^ ^25^ ^]^ Copyright 2011, Springer Nature. l) Schematic illustration of H_2_ dissociation and spillover at Pd/Cu single‐atom alloy (SAA). Reproduced with permission.^[^ ^34^ ^]^ Copyright 2012, AAAS. m) Ir_1_/FeO_x_ SAC for WGS reaction. Reproduced with permission.^[^ ^35^ ^]^ Copyright 2013, American Chemical Society. n) EXAFS data collected at different temperatures over 0.5 wt.% Pd/La‐Al_2_O_3_ for CO oxidation. Reproduced with permission.^[^ ^36^ ^]^ Copyright 2014, Springer Nature. o) Schematic illustration of Pt_1_/ZnO and Au_1_/ZnO SACs for methanol steam reforming (MSR). Reproduced with permission.^[^ ^37^ ^]^ Copyright 2014, American Chemical Society. p) Schematic illustration of the 3D network of MOF‐525‐Co. Reproduced with permission.^[^ ^38^ ^]^ Copyright 2016, Wiley‐VCH. q) Schematic illustration of the structure of Pt/HSC for oxygen reduction reaction (ORR). Reproduced with permission.^[^ ^39^ ^]^ Copyright 2016, Springer Nature. r) A computational study of Mo SAC for nitrogen fixation. Reproduced with permission.^[^ ^40^ ^]^ Copyright 2017, American Chemical Society. s) Schematic illustration of stable platinum dimers on graphene. Reproduced with permission.^[^ ^41^ ^]^ Copyright 2017, Springer Nature. t) Single‐atom Co—N_5_ site for CO_2_ selective reduction. Reproduced with permission.^[^ ^42^ ^]^ Copyright 2018, American Chemical Society. u) Schematic illustration of isolated Fe—N_4_ sites confined by N‐doped porous carbon support for mimicking two antioxidative enzymes of catalase (CAT) and superoxide dismutase (SOD). Reproduced with permission.^[^ ^43^ ^]^ Copyright 2019, Royal Society of Chemistry. v) Schematic illustration of carbon nanoframe‐confined Fe single‐atom sites with axial five‐N coordination for mimicking cytochrome P450. Reproduced with permission.^[^ ^44^ ^]^ Copyright 2019, AAAS. w) Pocketlike active site of Rh_1_/MoS_2_ SAC for selective crotonaldehyde hydrogenation. Reproduced with permission.^[^ ^45^ ^]^ Copyright 2019, American Chemical Society. x) Reaction pathways and energy graph of direct methane conversion (DMC) over Rh‐CeO_2_ NWs SAC. Reproduced with permission.^[^ ^46^ ^]^ Copyright 2020, Springer Nature. y) Ir Single Atoms for oxygen evolution reaction. Reproduced with permission.^[^ ^47^ ^]^ Copyright 2021, American Chemical Society. z) Cu_1_/ZSM‐5 SAC for direct methane oxidation. Reproduced with permission.^[^ ^48^ ^]^ Copyright 2021, Elsevier.

Based on previous experimental and theoretical foundations, the concept of SACs was first introduced by Zhang and co‐workers in 2011 when describing the high activity of single Pt atoms dispersed on FeO_x_ for CO oxidation.^[^ ^25^ ^]^ And then the extensive research on SACs explosively began (**Figure** **1**). Compared with traditional NP catalysts, not only do SACs have maximum atom utilization efficiency (100%) but also have well‐defined active centers, which provides an effective strategy to regulate the activity and selectivity in catalytic reactions.^[^ ^26^ ^]^ Moreover, SACs exhibit excellent performance in various chemical reactions due to the unique electronic states, geometric configuration, and coordination environment.

### 2.1 Concepts on Single‐Atom Active Sites

**Figure 2:**
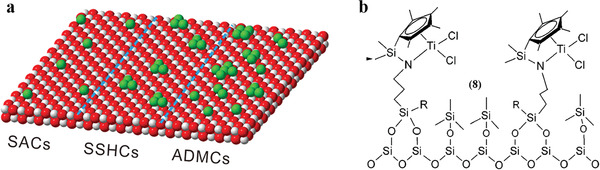
Schematic illustration of the various types of a) SAC, SSHC, ADSMC, and b) SIHC. (a) Reproduced with permission.^[^ ^54^ ^]^ Copyright 2020, American Chemical Society. (b) Reproduced with permission.^[^ ^55^ ^]^ Copyright 2004, American Chemical Society.

Before the concept of SACs was proposed in 2011, a few terms had been used to describe some related but distinct types of supported metal catalysts. We will discuss the following four specific academic terms: SAC, atomically dispersed supported metal catalyst (ADSMC), single‐site heterogeneous catalyst (SSHC), and site‐isolated heterogeneous catalyst (SIHC) as shown in **Figure** **2**.

SAC is defined as isolated metal atoms stabilized by the moieties on the appropriate supports.^[^ ^49^ ^]^ The active sites generally consist of single metal atoms as well as the direct neighboring atoms of the support surface or other functional species. The catalytic property of the individual active sites depends on the interactions between the single metal atoms and their neighboring atoms.^[^ ^50^ ^]^

ADSMC is defined as the catalysts with 100% metal dispersion. The metal species can be in the form of 2D rafts, small clusters, trimers, dimers, monomers, etc.^[^ ^30^, ^50^ ^]^ In fact, SAC can be considered as a subset of ADSMC though ADSMC is still used to describe SAC in many literatures nowadays.^[^ ^51^ ^]^ The structure and catalytic behavior of SAC are much better defined than those of ADSMC.^[^ ^50^ ^]^

As for SSHC, the “single site” (active center) probably consists of one or more atoms and each single site is spatially isolated from one another. For example, there is no spectroscopic or other cross‐talk between such sites. The energy of interaction between each site and a reactant is the same as every other single site. Besides, the structure of such sites is obviously characterized, similar to the single sites in homogeneous molecular catalysts.^[^ ^23^ ^]^ It's evident that when the single sites are single atoms, the definition of SSHC is equal to SAC.

SIHC commonly refers to a heterogeneous catalyst that contains spatially well‐separated organometallic complexes. The structure of organometallic complexes is well‐defined, and they are anchored to the surface of the support by ligands. The distinctive feature of SIHC is that all the active centers behave in the same manner, which are protected by ligands. SIHC with only one type of active center is usually regarded as SSHC.^[^ ^50^, ^52^ ^]^ Besides, SIHC and SSHC are frequently used universally in the reported literature. Some typical examples are highlighted in literature.^[^ ^53^ ^]^

In general, ADSMC, SSHC, and SIHC describe the dispersion of active sites from different perspectives. But these terms don't accurately describe the single‐atom active sites. The concept of SACs adequately reflects the homogeneity of single‐atom active sites.

### 2.2 Homogeneity on the Geometric Configuration of Single‐Atom Active Sites

Before starting the discussion on the homogeneous geometric configuration of single‐atom active sites, the heterogeneity in the geometric structures of NP/cluster catalysts will be briefly discussed. As NPs of different sizes and properties are bound to substrates, the nano‐catalysts display heterogeneous size, shape, and composition.^[^ ^56^ ^]^ The reactants and intermediates are adsorbed on uncertain sites of the surface of the NPs, such as edge, corner, terrace and kink sites, and metal‐support interface, thus forming different geometric structures and adsorption states on NPs and yielding low selectivity. Hence the active sites of supported nano‐sized catalysts are normally considered “poorly defined”.^[^ ^57^, ^58^ ^]^

**Figure 3:**
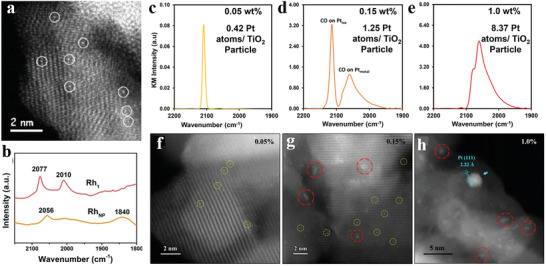
Geometric structure of SACs. a) Aberration‐corrected HAADF‐STEM images of the 0.03 wt.% Au_1_/FeO_x_ catalyst. Reproduced with permission.^[^ ^60^ ^]^ Copyright 2015, Springer Nature. b) DRIFT spectra of CO adsorption on Rh_1_/CeO_2_ and RhNP/CeO_2_ catalysts at RT. Reproduced with permission.^[^ ^61^ ^]^ Copyright 2020, Royal Society of Chemistry. c–e) IR spectra of CO adsorbed at saturation coverage and RT to 0.05 wt.% Pt/TiO_2_ SEA, 0.15 wt.% Pt/TiO_2_ SEA, and 1 wt.% Pt/TiO_2_ DI catalysts, respectively, which had been reduced at 240 °C in H_2_ prior to CO adsorption. f–h) Representative STEM images of the above 3 catalysts after reduction ex situ, where IR spectra in (c–e) correlate to STEM images of the same catalyst in (f–h). In the STEM images, Pt_iso_ species with a diameter of ≈175 pm are circled in yellow while clusters with a diameter ≈1 nm are circled in red. Reproduced with permission.^[^ ^59^ ^]^ Copyright 2017, American Chemical Society.

When metal species are downsized to single atoms dispersed on appropriate supports (forming SACs), all the metal atoms directly contact with the surface sites, which maximizes the number of interfacial atoms and makes all the metal atoms accessible to reactant molecules. Additionally, the metal species in SACs behave with high homogeneity on the geometric structure compared with the complicated surface atoms in NPs/clusters as demonstrated by HAADF‐STEM images (**Figure** **3**a) and the in situ CO‐DRIFTs (Figure 3b).

For example, 0.05 wt.% and 0.15 wt.% Pt/TiO_2_ catalysts prepared through strong electrostatic adsorption (SEA) wet impregnation technique, and 1 wt.% Pt/TiO_2_ catalyst prepared by incipient wetness (dry) impregnation method (donated as 0.05 wt.% Pt/TiO_2_ SEA, 0.15 wt.% Pt/TiO_2_ SEA, and 1 wt.% Pt/TiO_2_ DI catalysts, respectively) are compared to verify the homogeneity on the geometric configuration of single‐atom active sites and the heterogeneity on the geometric structures of NP/cluster catalysts (Figure 3c–h). The homogeneity of the geometric structures of isolated Pt atoms (Pt_iso_) species can be demonstrated by the predominant CO stretching band at 2112 cm^−1^ in the IR spectra that are assigned to CO adsorbed to Pt_iso_, with STEM image of 0.05 wt.% Pt/TiO_2_ SEA catalyst. While in the IR spectra of 0.15 wt.% Pt/TiO_2_ SEA catalyst, an increased relative intensity of the CO stretching band at 2040–2090 cm^−1^ assigned to metallic Pt cluster can be observed except for the CO stretching band at 2112 cm^−1^, compared to the 0.05 wt.% SEA catalyst. And the number of Pt clusters (0.84 nm average diameter) increases significantly based on the STEM image. In addition, the IR spectra of 1 wt.% Pt/TiO_2_ DI catalysts show almost exclusively CO stretching band intensity between 2040 and 2090 cm^−1^. And the STEM image shows the existence of predominantly clusters with an average diameter of 1.1 nm and a few larger particles with an average diameter of 4.3 nm. The geometric structures of NP/cluster catalysts are proved to be heterogeneous.^[^ ^59^ ^]^

Taking another instance, single Rh atoms anchored to the edges of 2D MoS_2_ sheets have been reported to generate a highly uniform HO‐Mo‐Rh_1_‐Mo‐OH configuration, resembling a pocketlike active center. The unique geometric structures of active sites possessing high homogeneity enable to restrict the adsorption configurations of bulky crotonaldehyde molecules and yield 100% selectivity.^[^ ^45^ ^]^

In short, the downsizing of metal particles enables the change of the geometric parameters like the population of interfacial atoms, lattice strain, bond length, and other physical/chemical parameters. Compared with NP catalysts, the SACs exhibit homogeneous geometric configurations.

### 2.3 Homogeneity on the Local Environments of Single‐Atom Active Sites

The coordination structure of the surface and interfacial metal species can not only generate both electronic and steric effects for optimizing their catalytic properties, but has been recently proved to be an important factor in many cases in tuning the catalytic pathway of supported metal catalysts.^[^ ^62^ ^]^ Arguably, when the metal species are downsized to atomic scale to form SACs, the considerable changes in the coordination structure, coordination number (CN), bond length/angle, and so on, significantly affect the selectivity/activity of the reaction, which relies on electron transfer and adsorption states.^[^ ^63^, ^64^ ^]^ For example, the Fe^3+^‐N‐C achieves high CO_2_ reduction activity (the FE_CO_ higher than 80% under the reduction potential from −0.2 to −0.5 V, and the current density of CO reached 20 mA cm^−2^ at −0.47 V). However, when the potential drops to −0.5 V or more negative, the change of coordination structure leads to a serious decrease in CO_2_ reduction activity. The N atoms coordinated with Fe atoms transformed from initial pyrrolic N into pyridinic N, accompanied by decreased CN from 4 to 3.^[^ ^65^ ^]^ Hence, it is vital to manipulate the coordination structure of the single‐atom catalytic metal centers.

**Figure 4:**
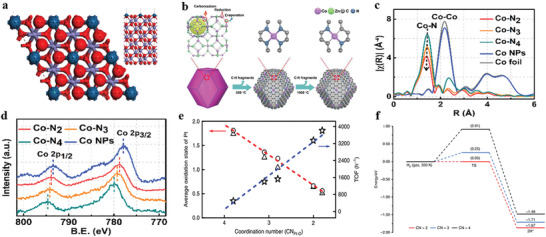
The coordination structures of SACs. a) Top and side view of the Fe_2_O_3_ (0001) O_3_‐termination hematite surface. Reproduced with permission.^[^ ^25^ ^]^ Copyright 2011, Springer Nature. b) Schematic illustration of forming Co‐N_4_ and Co‐N_2_. c,d) EXAFS and XPS spectra of Co atoms in Co NPs, Co‐N_2_, Co‐N_3_, and Co‐N_4_. Reproduced with permission.^[^ ^71^ ^]^ Copyright 2018, Wiley‐VCH. e) The relevance between activity and CN_Pt‐O_, the oxidation state of Pt. The star, triangle, and circle show TOF, the mean Pt oxidation state analyzed by XPS and XANES, respectively. f) Energy profiles for the dissociation of H_2_ on Pt_1_/Fe_2_O_3_‐T catalysts at 300 K. The black, blue, and red lines represent four‐coordinated Pt_1_/Fe_2_O_3_‐500, three‐coordinated Pt_1_/Fe_2_O_3_‐550 and two‐coordinated Pt_1_/Fe_2_O_3_‐600 surfaces, respectively. Reproduced with permission.^[^ ^72^ ^]^ Copyright 2019, Springer Nature.

For example, when single atoms are dispersed on various metal oxides, different M‐O(H) coordination will be formed. The single Pt atoms on various possible Fe_2_O_3_ surfaces constitute threefold hollow sites on the O_3_‐terminated surface, where each Pt atom is coordinated by three surface oxygen atoms to form a Pt‐O_3_ coordination structure (**Figure** **4**a). The surface Fe atoms of Fe_2_O_3_ (001) are considered to be partially substituted by Pt atoms, which have a larger size than that of Fe atoms, suggesting that the Pt atoms cannot fit in the plane with the other Fe atoms but are above the plane of O atoms.^[^ ^66^ ^]^ This configuration is highly accessible to the reactants and has higher stability in CO oxidation reactions.^[^ ^25^ ^]^ Atomically dispersed Pt^2+^ species are also successfully deposited at step sites of CeO_2_, which are preferred to be four‐coordinated inside the square pocket of O^2−^ ions at a nanofacet of CeO_2_.^[^ ^67^ ^]^ The Pt‐O_4_ coordination structure exhibits excellent activity and thermal stability. When the support is a non‐oxide, the stable coordination structure can still be formed through the interaction between metal species and support. The Pd‐N_4_ coordination geometry of Pd_1_/N‐graphene is known to be thermodynamically stable,^[^ ^68^ ^]^ which is explained by the strong coordination of Pd atoms by nitrogen, preventing the Pd aggregation. Hence, the Pd_1_/N‐graphene catalysts display excellent durability during selective hydrogenation of acetylene to ethylene.^[^ ^69^ ^]^

Furthermore, with the same binding atoms on supports, lowering the CN will change the electronic and geometric structure of single metal centers, thus influencing the performance of the reaction.^[^ ^70^, ^71^, ^72^ ^]^ For example, by regulating the CN of single Co atoms, CO_2_ electroreduction activity can be enhanced. It attempts to control the CN of single Co atoms (Co‐N_4_, Co‐N_3_, and Co‐N_2_) by varying the pyrolysis temperature (Figure 4b–d). The EXAFS data display the intensity of the Co‐N peak for Co‐N_2_ is much lower than that of Co‐N_3_ and Co‐N_4_, suggesting the decreased surrounding N number of Co center. Furthermore, with the decreased CN, the oxidation state of Co is also decreased, which results in more unoccupied 3*d* orbitals on Co atoms. Hence, the electronic structure of Co‐N_2_ benefits the low‐barrier formation and strong adsorption of CO_2_^•−^, inducing a higher CO_2_ reduction rate than Co‐N_4_. Moreover, the Co‐N_2_ sites enable CO formation turnover frequency (TOF) to reach a record value of 18 200 h^−1^, and further exhibit excellent performance in the efficient electroreduction of CO_2_.^[^ ^71^ ^]^ A similar conclusion can be drawn that the activity of Pt_1_/Fe_2_O_3_ is highly dependent on the CN of Pt‐O. The CN of Pt‐O not only has a linear relationship with the oxidation state but also displays an approximately linear relationship with the TOF, that is, the smaller CN of Pt‐O, the lower oxidation state of Pt single atoms, the higher the catalytic activity (Figure 4e). And both isotope experiments and density functional theory (DFT) calculations reveal the lower Pt‐O CN of the Pt_1_/Fe_2_O_3_, which is much easier for Pt single atoms to activate H_2_ molecules (Figure 4f), makes it several times more active in the hydrogenation of 3‐nitrostyrene.^[^ ^72^ ^]^

In short, adjusting the coordination structure of SACs can create both electronic and steric effects, which can affect the adsorption of reactants for optimizing their catalytic performance. The distinct atomic local environment of SACs is essential for determining superior catalytic performance in chemical reactions.

### 2.4 Homogeneity on the Electronic Configuration of Single‐Atom Active Sites

When downsizing to an atomically dispersed scale, the unsaturated coordination sites of isolated metal atoms present a flexible electronic environment to activate the catalyst and improve its catalytic activity.^[^ ^73^, ^74^, ^75^, ^76^, ^77^, ^78^ ^]^ It has been reported that the unoccupied 5*d* state of Pt_1_ single atoms in Pt_1_/Co_3_O_4_ can be tuned by electronic metal‐support interactions (EMSI), which modulates the adsorption energies of ammonia borane and H_2_, thus greatly leading to the high activity and stability for ammonia borane dehydrogenation catalysis.^[^ ^75^ ^]^ More importantly, downsizing the metal species enables to remarkably modulate the band gap, surface oxidation states, and other electronic states.^[^ ^79^ ^]^ The adjustment of electronic structure in SACs can substantially change the adsorption behavior of metal species for reactants, thereby affecting the reaction performance.

**Figure 5:**
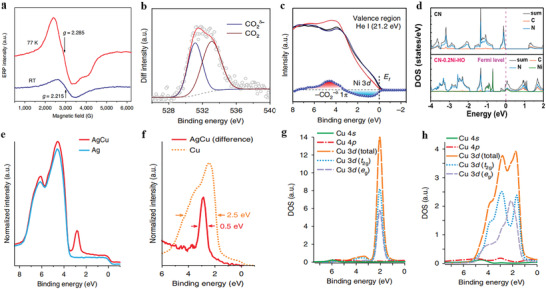
The electronic properties of the SACs. a) EPR spectra of A‐Ni‐NG measured at RT and 77 K. b) Variation in the XPS O 1*s* intensity caused by CO_2_ adsorption for A‐Ni‐NG. c) Valence band spectra of A‐Ni‐NG before (black line) and after (red line) CO_2_ gas exposure, and after desorption of CO_2_ by thermal treatment at 500  °C for 20 min in vacuum (dark blue line). The change in the valence band spectra of A‐Ni‐NG caused by CO_2_ adsorption is shown by the blue line. (a–c) Reproduced with permission.^[^ ^77^ ^]^ Copyright 2018, Springer Nature. d) Density of states of CN and CN‐0.2Ni‐HO. Reproduced with permission.^[^ ^78^ ^]^ Copyright 2020, Wiley‐VCH. e) The measured valence photoemission spectra (*hυ* = 150 eV) of an AgCu alloy that contained 0.3 at.% Cu and metallic Ag reveal the narrow Cu 3*d* states at a binding energy of ≈2.5 eV. f) The difference spectrum of AgCu and Ag, plotted with a Cu reference spectrum, demonstrate that the Cu 3*d* states in AgCu are one‐fifth the width they are in bulk Cu. g,h) Calculated Cu‐based pDOS of Ag_31_Cu_1_ (g) and pure bulk Cu (h) show that the Cu 3*d* states of AgCu are nearly degenerate, whereas they are heavily split in bulk Cu. The near degeneracy in AgCu indicates a very weak interaction between Cu 3*d* states and the surrounding Ag matrix. A. u., arbitrary units. (e–h) Reproduced with permission.^[^ ^80^ ^]^ Copyright 2018, Springer Nature.

For instance, the unique electronic configuration of Ni single atoms plays an important role in many important reactions.^[^ ^76^, ^77^, ^78^ ^]^ It has been revealed that the unpaired electrons in the 3*d* orbital are prone to be excited due to the unique 3*d*,^[^ ^9^ ^]^ S = ^1^/_2_ electronic configuration of the Ni single‐atom sites. In electrochemical CO_2_ reduction, the results prove that the delocalization of the unpaired electron in the Ni 3*d*_x2‐y2_ orbital facilitates the charge transfer from Ni atom to the carbon 2*p* orbital in CO_2_ to form a CO_2_^*δ*−^ species as shown in **Figure** **5**a–c. These processes lead to a higher Ni oxidation state and contribute to reducing the energy barrier for electrochemical CO_2_ reduction.^[^ ^77^ ^]^ Furthermore, the introduction of Ni single‐atom sites changes the density of states (DOS) of polymeric carbon nitride (CN) (Figure 5d). The DOS of CN presents the characteristics of semiconductors, where the electrons on the valence band are donated by N atoms and no electrons are distributed around the Fermi level. Based on the introduction of Ni single‐atom sites, Ni 3*d* electrons dominate the valence band and promote the conduction band crossing the Fermi level, resulting in the decreased band gap of the catalyst. The coordination of Ni single‐atom sites in the CN framework has effectively enhanced the visible‐light response, charge separation, and migration in the photocatalyst, indicating the electronic structure of Ni single‐atom sites is of great potential in elevating the photocatalytic performance.^[^ ^78^ ^]^

Atomically dispersed metal species can exhibit a unique free‐atom‐like electronic structure. The *d* band of single Cu atoms has been reported to become extremely narrow in AgCu alloys.^[^ ^80^ ^]^ The density of state (DOS) shows that the Cu 3*d* states are one‐fifth the width of the *d* band in bulk Cu as shown in Figure 5e–h, which clearly demonstrates the high homogeneity of single atoms in terms of electronic structure compared with that of metal NPs. The narrowness of the Cu 3*d* state causes strong coupling to the adsorbates and forms new covalent bonds. Furthermore, PDOS (partial density of state) calculations indicate an almost homogeneous coordination field for the Cu 3*d* states in Ag_31_Cu_1_, which indicates the solute's *d* states resemble those of a free atom. The electronic structure results in a localized negative charge on the Cu centers, leading to a stronger bonding to electronegative elements and a weaker bonding to electropositive elements (compared to elemental copper). Hence, the free‐atom‐like electronic structure of single Cu atoms in SAA has two main contributions: 1) ionic and 2) covalent.

In conclusion, the electronic structures (band gap, electronic DOS, surface oxidation states, etc.) can be significantly modulated when downsizing the NPs to single atoms. Especially, the homogeneity of the electronic structure of the metal sites has been significantly advanced at the atomic level compared with that of NPs, which obviously tunes the adsorption states of reactants and intermediates and correspondingly determines the catalytic performance of SACs.

### 2.5 Characterization of the Homogeneity of Single‐Atom Active Sites

The finding and characterization of SACs firmly are based on the employment of different characterization tools.^[^ ^51^ ^]^ Multitudinous sophisticated analytical techniques are necessary to characterize and identify the states (geometric, electronic configuration, and local environments) of single‐atom sites unambiguously.^[^ ^16^ ^]^ Meantime, the reacting behavior of substrates or intermediates at the active sites needs to be characterized as much as possible to understand the catalytic mechanisms of SACs. The advanced analytical tools like transmission electron microscopy (TEM),^[^ ^81^, ^82^, ^83^ ^]^ Fourier transform infrared spectroscopy (FTIR),^[^ ^84^, ^85^ ^]^ X‐ray absorption spectroscopy (XAS),^[^ ^86^, ^87^ ^]^ and X‐ray photoelectron spectroscopy (XPS)^[^ ^88^, ^89^ ^]^ have been combined for convincing conclusions,^[^ ^90^ ^]^ which means the information inferred from any characterization tool should be better demonstrated by other technique(s). Additionally, with the rapid development of operando characterization techniques, more and more in situ information on the active sites and reaction pathways can be obtained during the reaction process.^[^ ^91^, ^92^, ^93^ ^]^

**Figure 6:**
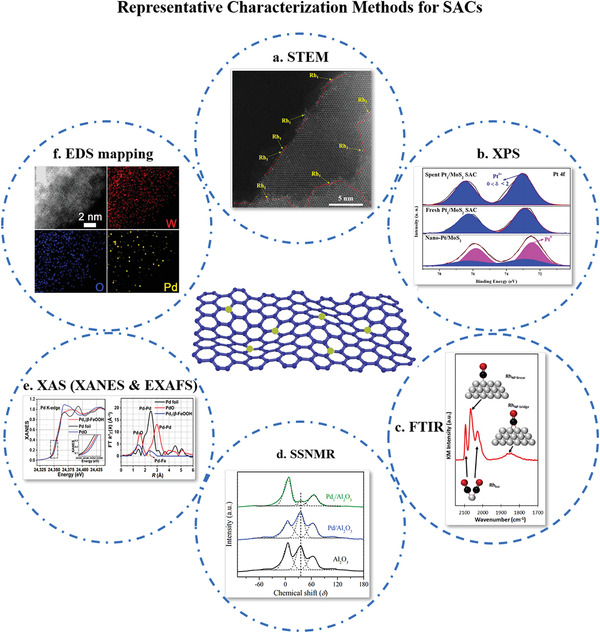
Representative Characterization Methods for SACs. a) Representative HAADF‐STEM image of Rh_1_/MoS_2_ SAC. Reproduced with permission.^[^ ^45^ ^]^ Copyright 2019, American Chemical Society. b) Pt 4*f* XPS spectra of spent Pt_1_/MoS_2_ SAC after five cycles, fresh Pt_1_/MoS_2_ SAC, and nano‐Pt/MoS_2_. Reproduced with permission.^[^ ^94^ ^]^ Copyright 2021, Wiley‐VCH. c) CO‐DRIFT spectrum adsorbed at 300 K on 4% Rh/TiO_2_. The ball‐and‐stick models show corresponding vibrational modes. Reproduced with permission.^[^ ^95^ ^]^ Copyright 2015, American Chemical Society. d) ^27^Al MAS NMR spectra (solid line) and their deconvolution results (short dot) of Pd_1_/Al_2_O_3_, Pd/Al_2_O_3_, and Al_2_O_3_. Reproduced with permission.^[^ ^96^ ^]^ Copyright 2020, Elsevier. e) Pd K‐edge XANES spectra and FT k^3^‐weighted Pd K edge EXAFS spectra of Pd foil, PdO, and Pd_1_/*β*‐FeOOH. Reproduced with permission.^[^ ^97^ ^]^ Copyright 2022, Elsevier. f) STEM‐EDS elemental mapping of Pd_1_/WO_2.72_. Reproduced with permission.^[^ ^98^ ^]^ Copyright 2021,Elsevier.

In this section, we will briefly introduce the important characteristics of commonly used and representative characterization techniques (**Figure** **6**).

#### 2.5.1 Scanning Transmission Electron Microscopy (STEM)

Scanning transmission electron microscopy (STEM) has been playing a crucial role in the direct imaging of catalytic centers of heterogeneous metal catalysts in the nanoscale resolution and even down to atomic resolution.^[^ ^99^ ^]^ It presents abundant direct information on the size and morphology of catalytic metal species on heterogeneous catalysts. Imaging atomic structures with sub‐angstrom resolution and identifying chemical species with single‐atom sensitivity are now routine for STEM. These advantages make significant contributions to the progress of catalysis research. Research for catalysts using these current microscopy techniques has deepened the understanding of atomic‐level catalytic mechanisms.^[^ ^90^ ^]^

For example, recently the dispersion of isolated Ni single atoms on carbon‐based materials has been verified by this technique.^[^ ^100^ ^]^ However, STEM is only locally focused and difficult to provide structural information of the whole sample.^[^ ^16^ ^]^ More details on probing the catalytical active centers by STEM can be found in some reviews.^[^ ^83^, ^101^ ^]^

#### 2.5.2 Fourier Transform Infrared (FTIR) Spectroscopy

FTIR has proven to be one of the most powerful basic techniques applied to the characterization of catalytic systems.^[^ ^32^ ^]^ It is a robust site‐specific characterization technique, which is used to distinguish and quantify the concentration of metal species in catalyst samples and provide insights into local geometry, stability, reactivity, and homogeneity of supported metal species.^[^ ^84^, ^102^, ^103^ ^]^ The IR absorption peak position reflects the molecular structure, which is applied to identify the structural composition or determine the chemical groups present; in addition, the absorption intensity can be used to quantitatively analyze the content of chemical groups. Particularly, with CO as a probe molecule (in situ CO‐DRIFTS), the catalytic reaction mechanism, oxidation states and geometric configuration of active sites in SACs can be characterized on the basis of changes in vibrational frequency and intensity of the probe modes.

One typical example is to characterize supported rhodium (Rh)‐group species.^[^ ^27^ ^]^ For single Rh sites, the position of CO adsorption peak changes according to the bonding mode of CO to Rh (Rh_1_(CO)_2_, bridged or atop) and the electronic structure of Rh.

#### 2.5.3 X‐Ray Absorption Spectroscopy (XAS)

The synchrotron radiation X‐rays characterization techniques are essential for a thorough understanding of the chemical state and structure of catalysts, which consists of the X‐ray absorption near‐edge structure (XANES) spectrum and the extended X‐ray absorption fine structure (EXAFS) spectrum. Since the valence electron distribution affects the nuclear electron energy, the XANES spectrum generally reflects the chemical valence states and electronic structure information of the measured elements. Through analyzing the fitting data of the EXAFS spectrum, true spatial distribution, the bonding conditions, and the coordination environment of the atoms can be inferred.^[^ ^99^, ^104^, ^105^ ^]^

The dynamic behaviors of both the geometric structure and chemical state of single atoms can be monitored by operando XAS under reactive atmosphere and conditions. This technique not only describes the stability of isolated atoms on supports but also provides important clues for further investigation of catalytic mechanisms.^[^ ^32^ ^]^ More importantly, the XAS technique mainly provides average structural information of all species present in the catalyst. However, precise information of isolated metal centers is difficult to obtain due to structural differences of active metal sites in many catalysts.^[^ ^16^ ^]^

#### 2.5.4 X‐Ray Photoelectron Spectroscopy (XPS)

XPS with high element sensitivity,^[^ ^106^, ^107^ ^]^ is widely applied to investigate the surface elemental composition as well as the electronic state of a specific element.^[^ ^108^, ^109^, ^110^ ^]^ It has been extensively employed to analyze the valence states of the metal centers in SACs. Recently, a series of quasi‐in situ XPS techniques have been proposed to characterize the evolution of the valence states of the catalysts and electron transfer behaviors of the active sites during the reaction process, which is essential for exploring the uniformity of active sites and reaction mechanism.^[^ ^111^, ^112^ ^]^ However, it's worth noting that the degree of vacuum in the reaction cell needs to be strictly limited in order to collect the emitted photoelectrons as the reaction proceeds. XPS is a powerful tool for detecting the surface reaction intermediates,^[^ ^90^ ^]^ but this technique can only detect the elements near the surface because of the limited detection depth.

#### 2.5.5 Solid‐State Nuclear Magnetic Resonance Spectroscopy (SSNMR)

Solid‐state NMR can reveal the chemical environment of the nucleus and the chemical bond/spatial connection network at the atomic and molecular levels.^[^ ^113^ ^]^ In addition to the aforementioned spectroscopic techniques, solid‐state NMR spectroscopy is also a powerful technique for the characterization of heterogeneous catalysts, especially magic angle spinning nuclear magnetic resonance (MAS NMR), making it a powerful tool to yield useful information on catalyst structure.^[^ ^114^, ^115^, ^116^, ^117^, ^118^ ^]^ NMR is used to confirm the anchoring site of single metal atoms and sometimes is supplementary of XAS. By employing ^27^Al MAS NMR spectroscopy, it is proposed that coordinatively unsaturated five‐coordinated Al^3+^ centers on the Al_2_O_3_ surface provide anchoring sites for single Pt or Ru atoms, playing a critical role in the formation of single‐atom metal sites on alumina supports.^[^ ^96^, ^119^ ^]^

#### 2.5.6 Energy Dispersive X‐Ray Spectroscopy Mapping (EDX Mapping)

Energy dispersive X‐ray spectroscopy mapping (EDX mapping), used to determine and quantify the spatial distribution of elements, is an extension of STEM to obtain atomic‐level chemical maps based on EDX spectroscopy.^[^ ^120^ ^]^ The technique is generally applied to identify the nanostructure (e.g., utilized in alloy systems for identifying complex intermetallic phases).^[^ ^121^, ^122^ ^]^ As HAADF‐STEM possibly provides insufficient information on samples, EDX mapping can provide local elemental characterization.^[^ ^123^ ^]^

When used for demonstrating the isolated metal atoms, EDX mapping is always combined with corresponding HAADF‐STEM images. For instance, the isolated dispersion of Cu species during the whole reaction process and the homogeneous dispersion of C, N, S, and Cu atoms in the catalyst can be confirmed by EDX mappings.^[^ ^124^ ^]^

Other useful techniques in heterogeneous catalysis research are widely used in studying SAC properties. One may want to read the related literature to find more details on the characterization of the homogeneity of single‐atom active sites.^[^ ^125^, ^126^ ^]^

### 2.6 Exploring Uniformity of Active Sites under Reaction Condition

Structural evolution of active sites can occur during pre‐treatment and reaction processes on isolated metal atoms, NPs, and clusters.^[^ ^127^, ^128^, ^129^ ^]^ Two mechanisms, Ostwald ripening and migration‐coalescence process, have been proposed to expound the sintering and agglomeration of metal NPs, which may occur simultaneously during the agglomeration process of the catalysts under the real reaction conditions.^[^ ^130^, ^131^ ^]^

For SACs, although thermal‐stable catalysts are successfully fabricated, the geometric configuration of the metal sites may still be in the dynamic changes during the catalytic cycles. Harsh reaction conditions (such as high temperature) and oxidative/reducing reactants (e.g., CO, NO, H_2_, and O_2_) can affect or induce the agglomeration, and redispersion of supported single atoms.^[^ ^132^, ^133^ ^]^ Obviously, the dynamic changes in SACs under reaction conditions will change the uniformity of active sites.^[^ ^56^ ^]^ Therefore, it's necessary to understand the evolution of uniformity of active sites under reaction conditions and appropriate strategies should be taken to improve the stability and uniformity of SACs.^[^ ^66^ ^]^ Here, we provide several examples that demonstrate the stability/instability and uniformity of SACs in various chemical transformations.

The remarkable development of advanced characterization techniques (e.g., aberration‐corrected electron microscopy) enables the researchers to understand the dynamic evolution of the structure of active sites during the reactions.

Some catalyst systems are sufficiently stable throughout the reactions.^[^ ^60^ ^]^ Taking Pd_1_/CeO_2‐x_^135^ as an example, the oxygen vacancies in CeO_2‐x_ nanorods work as anchoring sites for restricting and stabilizing the Pd atoms because of strong metal‐support interaction (SMSI). Characterization results of Pd_1_/CeO_2‐x_ catalyst after recycling tests in the hydrogenation of cinnamaldehyde underscores the robustness of completely dispersed Pd single atoms supported on CeO_2‐x_ nanorods. Correspondingly, the SACs show superb stability in conversion and selectivity in recycling tests.

**Figure 7:**
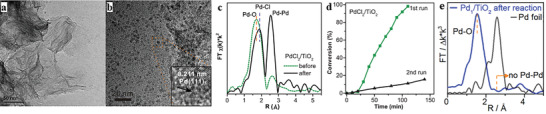
Stable Pd‐TiO_2_ and instable PdCl_2_/TiO_2_ catalysts. a,b) Typical TEM images of PdCl_2_/TiO_2_ after hydrogenation reaction. c) FT‐EXAFS spectra at the Pd K‐edge of PdCl_2_/TiO_2_ before and after hydrogenation reaction. d) First‐and second‐run catalytic results of PdCl_2_/TiO_2_. e) FT‐EXAFS spectra of Pd_1_/TiO_2_ at the Pd K‐edge after hydrogenation reaction. Reproduced with permission.^[^ ^135^ ^]^ Copyright 2016, AAAS.

Another stable atomically dispersed Pd‐TiO_2_ catalyst synthesized through a photochemical route, is utilized in styrene hydrogenation.^[^ ^135^ ^]^ Inspiringly, its reaction rate is still maintained even after 20 catalytic cycles. Besides, no change is detectable in the EXAFS fitting curves (**Figure** **7**e) after 20 cycles. These results suggest the robust and stable structure of atomically dispersed Pd_1_/TiO_2_ under reaction conditions. Interestingly, the researchers also prepare another catalyst (denoted PdCl_2_/TiO_2_) through the same method as for Pd_1_/TiO_2_ but without the UV treatment. No Pd‐Pd coordination is detected in the fresh PdCl_2_/TiO_2_, and Pd atoms are in the form of PdCl_2_ species anchored on TiO_2_. When utilizing under the same reaction conditions, the reaction rate of PdCl_2_/TiO_2_ already declines during the first cycle (Figure 7d) and keeps decreasing after every recycle. It is revealed that Pd‐Pd bonds emerge for the PdCl_2_/TiO_2_ catalyst in EXAFS studies (Figure 7c), with fine metallic Pd NPs observed in TEM images of the catalyst (Figure 7a,b). Notably, the existence of Pd‐Cl bonds has a deleterious effect on the catalysis, which destabilizes isolated Pd single atoms over TiO_2_ and induces their sintering into NPs during catalytic transformations. Therefore, to prepare highly stable and active Pd catalysts, it appears important to remove the Cl^−^ ligands on Pd under mild UV conditions.

The structural changes in Au species of low‐loading (<1 wt.%) Au‐CeO_2_ catalysts have been also tracked in WGS reaction tests.^[^ ^136^ ^]^ The Au‐Au CN is zero in the fresh 0.5AuCe(La)O_x_ sample, suggesting that Au is atomically dispersed in cerium oxide. It can be seen that the Au‐Au CN is stabilized at 6.5±2.4 after the WGS reaction at 100 °C. Even though zerovalent Au is found in used catalysts at 100 °C by the XANES spectra, gold particles are not found and the gold clusters in this material should not be larger than 1nm. In another experiment, the newly prepared catalyst is loaded into the polyimide reactor and the WGS reaction is operated at 200 °C. The CN of Au‐Au is 8.7±1.5 in the obtained sample, higher than that of the used sample at 100 °C. STEM/EDX results of the used sample at 200 °C suggest the Au concentration of selected 3 spots is a little higher than the mean concentration, which indicates the agglomeration of Au. The catalytic activity is reflected by CO conversion at each temperature and the entirely dispersed Au‐O‐Ce fresh sample reaches the maximum activity of Au‐CeO_2_.

**Figure 8:**
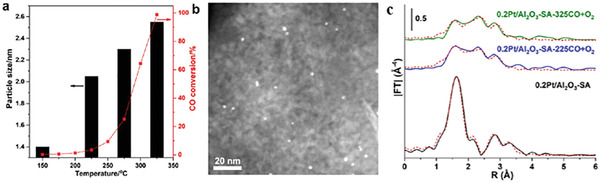
Catalytic and characterization results of 0.2Pt/Al_2_O_3_‐SA catalysts after structural transformation. a) Catalytic performance and the structural evolution of the 0.2Pt/Al_2_O_3_‐SA sample for CO oxidation under reaction conditions. b) Representative HAADF‐STEM image of the 0.2Pt/Al_2_O_3_‐SA sample after the CO oxidation reaction. c) EXAFS spectra (not phase‐corrected) of the fresh 0.2Pt/Al_2_O_3_‐SA sample and the corresponding sample after the CO+O_2_ reaction at 225 and 325 °C, respectively. The dashed red curves are the fit curves of the EXAFS spectra. Reproduced with permission.^[^ ^137^ ^]^ Copyright 2019, American Chemical Society.

The evolution of Pt single atoms loaded on Al_2_O_3_ during the CO oxidation process is investigated.^[^ ^137^ ^]^ According to the EXAFS spectra (**Figure** **8**c), a new contribution at 2.7 Å related with Pt‐Pt coordination suggests the transformation of atomically dispersed Pt species into agglomerated Pt clusters and NPs. The agglomeration of highly dispersed Pt atoms can also be demonstrated from the STEM images of 0.2Pt/Al_2_O_3_‐SA catalyst (Figure 8b) after CO + O_2_ reaction from 150 to 325 °C. Moreover, when the reaction temperature is increased from 150 to 325 °C, the in situ formed Pt NPs become bigger (Figure 8a). It's obvious that the Pt species undergo dynamic structural transformation in CO oxidation reaction.

As discussed above, single‐atom active sites may show dynamic structural transformations during the reaction in varieties of reactions. Understanding the evolutional behavior of SACs under reaction conditions helps to stabilize the uniformity of active sites and thus provides insights into developing stable SACs for practical applications.

### 2.7 Synthetic Methods for SACs

**Figure 9:**
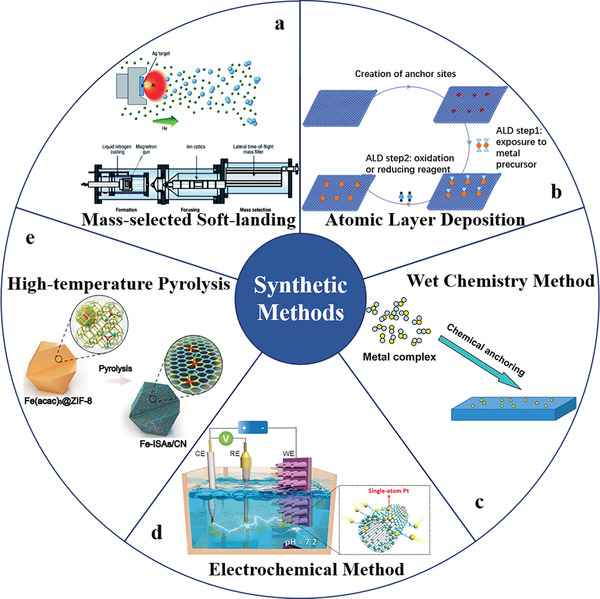
Commonly used synthetic methods for SACs. a) Production of size‐selected cluster beams. Reproduced with permission.^[^ ^139^ ^]^ Copyright 2003, Springer Nature. b) Schematic illustration of ALD. Reproduced with permission.^[^ ^138^ ^]^ Copyright 2018, Royal Society of Chemistry. c) Schematic illustration of wet chemistry method. Reproduced with permission.^[^ ^12^ ^]^ Copyright 2013, American Chemical Society. d) Schematic illustration of the potential‐cycling synthesis process. Reproduced with permission.^[^ ^140^ ^]^ Copyright 2017, Wiley‐VCH. e) Schematic illustration of the formation of Fe‐ISAs/CN by High‐temperature pyrolysis. Reproduced with permission.^[^ ^141^ ^]^ Copyright 2017,Wiley‐VCH.

When metals are dispersed to the atomic level, single atoms have high surface energy, which makes the monoatomic catalyst easy to aggregate during the synthesis process. Therefore, the synthesis of SACs and maintaining the high dispersion of metal atoms under the reaction conditions are still a huge challenge.^[^ ^138^ ^]^ At present, the synthesis methods of SACs mainly include mass selected‐soft landing method, atomic layer deposition (ALD), wet chemistry method, high‐temperature pyrolysis, electrochemical method, etc (**Figure** **9**).

#### 2.7.1 Mass‐Selected Soft‐Landing

The mass‐selected soft‐landing method has high selectivity, but can only be used in an ultra‐high vacuum with low yield. The pure metal solid source is heated to a high temperature, and the metal atoms are selectively vaporized by inert gas in the mass filter, in which the number of atoms in the cluster can be controlled by the mass. Finally, the selected atoms fall gently on the host.^[^ ^142^ ^]^ However, the development of this method is hindered by harsh synthesis conditions and extremely low yield, which makes it not suitable for large‐scale production of SACs. Moreover, the SACs prepared by this method are usually supported on the hosts by physical adsorption without forming a strong covalent bond or coordination bond with the support, which makes these single atoms easy to aggregate in the subsequent reaction.

#### 2.7.2 Atomic Layer Deposition

ALD is a kind of thin film deposition technology based on the gas‐solid reaction between gas and carrier, which can be used to synthesize highly controlled SACs by adjusting reaction conditions (including temperature and pressure).^[^ ^143^, ^144^, ^145^ ^]^ In the process of synthesizing SACs by the ALD method, the self‐limiting property of ALD allows the catalyst materials to be uniformly deposited on the catalyst support with a high specific surface area. In recent years, ALD has been used to uniformly deposit metal species on oxides or soft materials.^[^ ^146^, ^147^ ^]^ For instance, single Pt atoms are dispersed on CeO_2_, Co_3_O_4_, ZrO_2_, and graphene through the ALD method.^[^ ^75^ ^]^ Compared with other methods, this method can control the synthesis of SACs better due to the self‐limiting reaction on the scaffold surface. However, the ALD method also has some disadvantages, such as harsh reaction conditions, limited applicability, and high cost.

#### 2.7.3 Wet Chemistry Method

The wet chemistry method is a kind of conventional method for the preparation of SACs, which is divided into coprecipitation and impregnation. The coprecipitation method is to add a precipitator to the solution containing two or more cations, and then obtain SACs with active species uniformly dispersed.^[^ ^148^ ^]^ In this way, the reaction condition is simple and easy to operate. However, in the process of coprecipitation reaction to generate precipitation, the single atoms are not only loaded on the surface of the carrier, but also inside the carrier, resulting in low atomic utilization. The impregnation method is one of the classic methods for the preparation of the heterogeneous catalyst. In the impregnation process, the support is contacted with the metal salt solution, which is then adsorbed or stored on the surface or pore structure of the support. And after the processes of separation, drying, and calcination, the catalyst is prepared.^[^ ^149^ ^]^ The challenge of this method is how to increase the loading rate of metal single atoms.^[^ ^150^ ^]^

#### 2.7.4 Pyrolysis Method

Pyrolysis method is a widely used technology developed in recent years to synthesize high‐loading SACs in situ by decomposing precursors at elevated temperatures under gas atmosphere (e.g., N_2_, NH_3_, and Ar).^[^ ^151^, ^152^ ^]^ The precursors include hybrids of metal salts, metal complexes or metal oxides and various carbon substrates,^[^ ^151^, ^152^, ^153^, ^154^, ^155^, ^156^ ^]^ MOFs,^[^ ^141^, ^157^ ^]^ polymers,^[^ ^158^, ^159^ ^]^ etc. A series of SACs are synthesized by complexing metal cations with 1,10‐phenanthroline and decomposing the complexes absorbed on carbon black at 600 °C under an argon atmosphere.^[^ ^160^ ^]^ When using MOFs as the substrates, nanocages with high concentration, specific surface area, and anchoring sites benefit the preparation of stable SACs. Besides, reasonably designing the adjustable structure of MOFs allows generalizing this method to various metals that are atomically dispersed on supports.^[^ ^157^ ^]^ Li and co‐workers have designed a type of Zn/Co bimetallic MOF, where the Zn^2+^ sites replace a percentage of Co^2+^ sites. Then Zn atoms can be evaporated away over 800  °C, and Co ions are reduced in situ by carbonized organic linkers.^[^ ^157^ ^]^ Moreover, three different Co SACs with Co‐N_4_, Co‐N_3_, and Co‐N_2_ coordination structures are selectively prepared through the same strategy at 800, 900, and 1000  °C, respectively.^[^ ^71^ ^]^ Besides, sacrificial templates, like MgO, SiO_2_, and so on, are introduced during the pyrolysis process to obtain SACs with higher BET surface area.^[^ ^161^, ^162^ ^]^ The advantages of this pyrolysis method are simple operation and possible utilization of inexpensive and abundant raw materials.^[^ ^163^ ^]^ However, high‐temperature conditions and using toxic corrosive agents (HF, etc.) are disadvantages of this strategy.

#### 2.7.5 Electrochemical Method

This method allows the metal atoms to directly disperse on the electrode substrate (for a two‐electrode system, metal atoms are deposited on the cathode or anode by adapting deposition potentials or deposition time).^[^ ^164^ ^]^ SACs synthesized through this approach can be directly used in the corresponding electrocatalytic reaction without an adhesive. A universe electrochemical deposition approach is developed through cathodic or anodic deposition that endows the SACs with different electronic states.^[^ ^164^ ^]^ Besides, an electrochemical potential window strategy is proposed, which uses electrochemical oxidation to leach out the aggregate metal species on the support while the strongly bound single atoms remain at the substrate.^[^ ^165^ ^]^

In addition to the above methods, there are some other strategies developed to prepare SACs such as atom trapping,^[^ ^166^ ^]^ molten‐salt‐mediated method,^[^ ^167^ ^]^ photochemical deposition,^[^ ^168^ ^]^ etc. It is vital to stabilize single metal atoms during the synthesis process and under subsequent reaction conditions because isolated metal atoms incline to agglomerate to NPs. Combined with advanced characterization techniques, synthetic methods will continuously develop to fabricate highly stable SACs on a large scale towards industrial manufacture.

### 2.8 Stabilizing Uniformity of Active Sites under Reaction Condition

Efforts are required to develop effective approaches for improving the reactive stability of SACs while achieving excellent catalytic performance. During the past few decades, massive researches on the synthetic strategy for stabilizing SACs have been performed and SMSI is considered to be the essential factor for preventing single atoms from aggregation.^[^ ^50^, ^169^, ^170^, ^171^ ^]^ The strength of the interaction can modulate the interface charge transfer, the electronic structure of the metal, and so on, which in turn influences the selectivity, conversion, and stability of SACs.^[^ ^172^, ^173^ ^]^ Hence, the anchoring effect of support to metal atoms should be sufficient to stabilize the uniformity of active sites under diversified reaction conditions. Especially, the selection of supports is extremely important in regulating the interactions and optimizing the dispersion of single metal atoms.^[^ ^174^ ^]^

**Figure 10:**
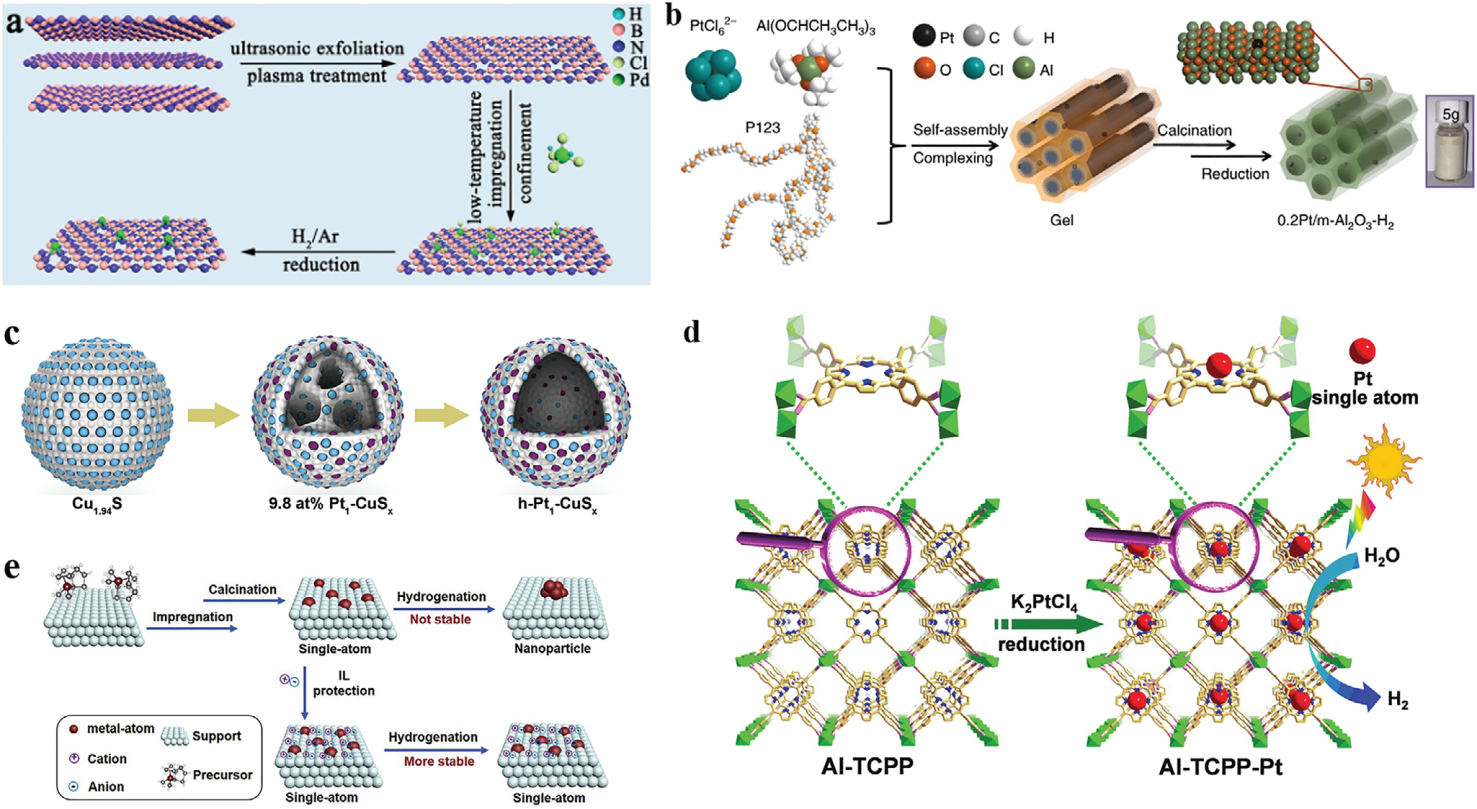
Examples of stabilizing uniformity of active sites under reaction conditions. a) Schematic illustration of the synthesis of Pd_1_/h‐BN. Reproduced with permission.^[^ ^179^ ^]^ Copyright 2021, American Chemical Society. b) Schematic illustration of the fabrication of 0.2Pt/m‐Al_2_O_3_‐H_2_. Reproduced with permission.^[^ ^186^ ^]^ Copyright 2017, Springer Nature. c) Schematic illustration of the structure evolution of h‐Pt_1_‐CuS_x_. The Cu, Pt, and S atoms are presented as blue, purple, and white balls, respectively. Reproduced with permission.^[^ ^188^ ^]^ Copyright 2019. Elsevier. d) Schematic illustration of the fabrication of Al‐TCPP‐Pt for photocatalytic hydrogen production. Reproduced with permission.^[^ ^191^ ^]^ Copyright 2018, Wiley‐VCH. e) Schematic illustration of the synthesis of SACs and the stabilization by ILs. Reproduced with permission.^[^ ^192^ ^]^ Copyright 2019. Elsevier.

Metal‐support interactions (MSI) can be improved by selecting an appropriate substrate, and thus the single atoms can be stabilized to a certain extent. One approach to enhance the stability of single atoms is surface defects engineering strategy.^[^ ^175^, ^176^, ^177^ ^]^ The appearance of defects can change the surrounding electronic structure and coordination environment, resulting in the presence of vacancies and unsaturated coordination sites, and thus enhance the binding strength with metal precursors while capturing them.^[^ ^178^ ^]^ For instance, Pd_1_/h‐BN is created by anchoring isolated Pd single atoms on a defective hexagonal boron nitride (h‐BN) nanosheet (**Figure** **10**a).^[^ ^179^ ^]^ The DFT calculations confirm that the B vacancy can provide stable anchoring sites for Pd atoms, which contributes to the excellent stability. Tested in the hydrogenation of cinnamaldehyde, Pd_1_/h‐BN shows excellent catalytic performance. The selectivity toward hydrocinnamaldehyde is 93% and the conversion reaches 99% within 180 min, along with the TOF of 1112 h^−1^. It's also confirmed that no Pd leaching. Moreover, the characterization results of Pd_1_/h‐BN tracking the recycling test highlight its excellent stability since no obvious evidence is collected for the formation of Pd aggregation.

Another strategy is to spatially confine single metal atoms into pores or molecular‐scale cages within porous materials (e.g., zeolites and metal‐organic frameworks (MOFs)),^[^ ^48^, ^180^, ^181^, ^182^, ^183^ ^]^ which encapsulate single metal atoms to avoid migration and agglomeration.^[^ ^184^ ^]^ Typically, spatial confinement strategy consists of two steps: 1) using molecular pores of porous materials as cages to trap and anchor mononuclear metal precursors to achieve uniform spatial distribution and atomic dispersion; 2) following by post‐treatment to get rid of ligands of precursors and obtain single metal atoms firmly anchored in the frameworks of supports.^[^ ^185^ ^]^ Taking Pt/m‐Al_2_O_3_^188^ as an example, the Pt single atoms are stabilized in the internal surface of mesoporous Al_2_O_3_ (Figure 10b). The stability of 0.2Pt/m‐Al_2_O_3_‐H_2_ catalyst is investigated for selective hydrogenation of 1,3‐butadine. To assess the long‐term stability, the catalyst should endure the mixture of gas reagents at 200 °C for 24 h, and then its catalytic performance is re‐evaluated at 30 °C for 12 h. Notably, the activity of 0.2Pt/m‐Al_2_O_3_‐H_2_ is slightly increased and the selectivity towards butenes nearly reaches 99%, demonstrating the stabilization of Pt active sites even after high‐temperature treatment. Besides, abundant well‐separated Pt single atoms are observed by HAADF‐STEM on spent samples and no evident Pt atom aggregation is identified. In the recycling experiment (100–400 °C), its activity increases marginally in the first 13 cycles, and becomes very steady in the subsequent 37 rounds for CO oxidation. The temperature is then maintained at 400 °C for 220 h and the CO conversion is kept 100% during the whole process. Later, the spent catalyst is tested again in recycling experiments (100–400 °C) and the conversion‐temperature profiles exactly resemble those obtained before long‐term treatment at 400 °C. Finally, the temperature is set at 230 °C for 70 h and the CO conversion has no appreciable drop. No Pt NPs are observed from the TEM image and TEM‐EDS elemental mapping, suggesting that Pt species are atomically dispersed on spent 0.2Pt/m‐Al_2_O_3_‐H_2_ catalyst. And Pt‐O contribution remains as the only prominent shell, powerfully demonstrating that Pt species mainly maintains single‐atom identity.

The migration and agglomeration of single atoms can also be prevented by introducing atoms with lone pairs of electrons (such as N, P, and S) onto the support since the strong interaction between those nonmetal atoms and single atoms will trap the individual metal atoms tightly.^[^ ^187^ ^]^ The h‐Pt_1_‐CuS_x_ catalysts are fabricated through the atomical dispersion of Pt atoms in a special hollow CuS_x_ support (Figure 10c).^[^ ^188^ ^]^ The strong Pt‐S interaction ensures the preferential coordination between Pt and S atoms and the CN is around 4. It's proved that the synthesized h‐Pt_1_‐CuS_x_ catalyst is stable for the H_2_O_2_ generation reaction as the decline of selectivity is slight after durability tests. The bright bots in AC‐HAADF‐STEM image after durability tests represent the isolated Pt single atoms in the catalysts, and there is no Pt cluster. The strong interaction between Pt and S accounts for the high stability of the catalyst.

In addition to conventional nonmetal species doping, some porous materials with abundant heteroatoms such as N and O can perform as coordination atoms to capture isolated metal species for their nature of lone pairs of electrons.^[^ ^189^, ^190^ ^]^ Fang and co‐workers confine Pt single atoms into a highly stable Al‐based porphyrinic MOF (donated as Al‐TCPP),^[^ ^191^ ^]^ in which the Pt atoms are implanted into the center of porphyrin linkers in Al‐TCPP (Figure 10d) and strongly interact with pyrrolic N atoms. Experiment results show that the photocatalytic hydrogen production rate doesn't change over Al‐TCPP‐0.1Pt during the four catalytic cycles. No particle is observed in the TEM and HAADF‐STEM images, revealing the atomically dispersed Pt species on Al‐TCPP without aggregation. Moreover, its well‐retained crystallinity and structural integrity are demonstrated by powder XRD after catalytic recycles, further indicating the excellent stability under reaction conditions.

Except for a suitable choice of support, it is proposed that the introduction of ionic liquids may be a feasible method to strengthen the stability of SACs. According to Ding's study, the coating process of ILs on Pt is facile and three ILs are employed.^[^ ^193^ ^]^ Both 0.2Pt_1_/HAP and ILs‐0.2Pt_1_/HAP are utilized for propylene hydrogenation at 90 °C for 1 h.^[^ ^192^ ^]^ It can be observed from the HAADF‐STEM images of spent catalysts that Pt NPs form on 0.2Pt_1_/HAP. By contrast, singly dispersed Pt species are only identifiable on BmimTf_2_N‐0.2Pt_1_/HAP. Furthermore, EXAFS shows that BmimTf_2_N‐0.2Pt_1_/HAP displays a dominant peak of Pt‐O coordination. However, 0.2Pt_1_/HAP exhibits a strong Pt—Pt contribution. All these results demonstrate that the ILs shield Pt single atoms and provide electrostatic stabilization against aggregation (Figure 10e).

## 3 Selective Hydrogenation over SACs

### 3.1 Selective Hydrogenation of Nitroaromatic Hydrocarbons

Aromatic amines are key intermediates for the production of fine chemicals like agrochemical, pharmaceutical, and dye compounds, which can be synthesized by the reduction of corresponding nitroaromatic hydrocarbons.^[^ ^195^, ^196^ ^]^ Some simple nitroarene molecules like nitrobenzene can be reduced easily through catalytic hydrogenation over Group VIII metal catalysts.^[^ ^197^ ^]^ In fact, most of the nitroaromatic hydrocarbons (e. g. 3‐nitrostyrene) have other reducible groups, such as ‐C=C, ‐C≡C, ‐C=O, ‐CN, etc. Therefore, selective hydrogenation of the ‐NO_2_ groups without reducing other reducible groups is a desirable but challenging task.^[^ ^198^ ^]^ In the past decades, many attempts have been made to develop efficient and selective catalysts for the selective hydrogenation of nitroaromatic hydrocarbons.^[^ ^199^, ^200^, ^201^, ^202^, ^203^, ^204^, ^205^, ^206^, ^207^ ^]^ Herein, we summarize some recent advances in selective hydrogenation of nitroaromatic hydrocarbons catalyzed by SACs in this section. The related references on the selective hydrogenation of nitroaromatic hydrocarbons over NPs‐based catalysts can be found in the previous literature.^[^ ^208^, ^209^, ^210^ ^]^

Noteworthy, Pt single atoms especially prefer the adsorption of ‐NO_2_ groups while the selectivity for C=C double bonds drops.^[^ ^211^ ^]^ Wei and co‐workers report FeO_x_‐supported Pt single‐atom and pseudo‐single‐atom structures are extremely active, chemoselective, and recyclable for the hydrogenation of a variety of functionalized nitroarenes.^[^ ^148^ ^]^ For hydrogenation of 3‐nitrostyrene, the TOF of the SACs and pseudo‐SACs reaches as high as ≈1500 h^−1^, and the selectivity toward 3‐aminostyrene is approximately 99% under mild conditions. The strong interaction between single or pseudo‐single atoms of Pt and the FeO_x_ support contributes to the superior performance of these catalysts. As a result, significant electrons transfer from the Pt species to the FeO_x_ support. Specifically, the presence of isolated and positively charged Pt centers, as well as the appropriately reduced metal oxide surfaces, favor the preferential adsorption of ‐NO_2_ groups, yielding significantly enhanced catalytic performance.

**Figure 11:**
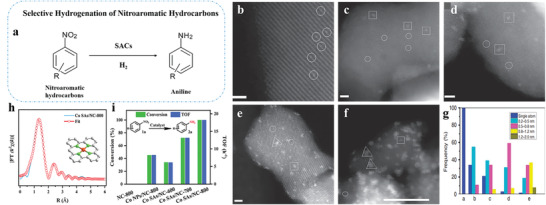
Selective hydrogenation of nitroaromatic hydrocarbons. a) Schematic illustration. HAADF‐STEM images of b) 0.08%Pt/FeO_x_‐R200, c) 0.08%Pt/FeO_x_‐R250, d) 0.31%Pt/FeO_x_‐R250, e) 0.75%Pt/FeO_x_‐R250, and f) 4.30%Pt/FeO_x_‐R250. The single atoms, 2D rafts, and 3D clusters/particles are marked by circles, squares, and triangles in the images, respectively. g) Graph of size distributions. Reproduced with permission.^[^ ^148^ ^]^ Copyright 2014, Springer Nature. h) EXAFS fitting and i) catalytic results of Co SAs. Reproduced with permission.^[^ ^194^ ^]^ Copyright 2021, Wiley‐VCH.

In contrast to the 0.08%Pt/FeO_x_ SACs and pseudo‐SACs, when the Pt loading is between 0.31 and 4.30 wt.%, the activity and selectivity of resultant catalysts are declined with the increase of the Pt loading when the reduction temperature is 250 °C. The reason for the decreased activity and selectivity is that 3D particles bigger than 1 nm in diameter emerge where Pt‐Pt metallic bonding forms (**Figure** **11**b–g). The presence of particles not only reduces the accessibility of Pt atoms, but also accelerates the rate of the side reaction C=C hydrogenation.

When Pt single atoms are embedded on the surface of Ni nanocrystals, the prepared Pt_1_/Ni nanocrystals catalysts are highly active and selective for the hydrogenation of 3‐nitrostyrene.^[^ ^26^ ^]^ The TOF of Pt_1_/Ni nanocrystals with different Pt contents is ca. 1800 h^−1^, higher than that of Pt clusters/Ni nanocrystals (865 h^−1^). The researchers further explore the catalytic selectivity in the hydrogenation of 3‐nitrostyrene. Only ‐NO_2_ groups are hydrogenated on Pt SACs, with the selectivity of > 99% for 3‐vinylaniline. For Pt clusters/Ni nanocrystals, both ‐NO_2_ groups and C=C bonds are concurrently hydrogenated. After 100 min, the selectivity for 3‐vinylaniline is only 19% for Pt clusters/Ni nanocrystals. Mechanistic studies reveal that H_2_ is spontaneously dissociated on both Pt and Ni atoms and H atoms diffuse facilely on Pt_1_/Ni nanocrystals, which bring about sufficient hydrogen supply and the remarkable activity of Pt_1_/Ni nanocrystals. Besides, the active sites in Pt_1_/Ni nanocrystals are composed of Pt single atoms and neighboring Ni atoms, which make the adsorption configuration of 3‐nitrostyrene favorable for the activation of ‐NO_2_ groups, clarifying the high selectivity of 3‐vinylaniline.

Currently, SACs of non‐noble metal loaded on nitrogen‐doped carbon support are being extensively studied for selective hydrogenation of aromatic compounds.^[^ ^212^ ^]^ A Co SAC supported on N‐doped carbon (Co SAs/NC) is prepared by pyrolysis of a Zn/Co bimetallic zeolitic imidazolate framework, Zn_24_Co_1_‐BMOF. The catalysts exhibit excellent performance in the selective hydrogenation of nitrobenzene, with ≈100% conversion of nitrobenzene and >99% selectivity to aniline after 4h.^[^ ^213^ ^]^ In comparison, the Co NPs supported on NC (Co NPs/NC) give lower activity, with 84.8% conversion of nitrobenzene and 97.4% selectivity to aniline at 8h. And the catalytic activity of Co SAs/NC is 5.4 times higher than that of Co NPs/NC. The analysis of characterization results of XRD, TEM, HAADF‐STEM, XPS, and XAFS reveal that atomically dispersed Co species and N‐doped carbon support play an important role in obtaining the high activity and selectivity of Co SAs/NC. Researchers put forward the doped N atoms in carbon support not only enable to decrease the dissociation energy of H_2_^[^ ^216^ ^]^ but also favor the preferential adsorption of ‐NO_2_ groups by affecting the surface basicity and polarity of the catalyst.^[^ ^215^ ^]^

Co SAs/NC‐800 (the pyrolysis temperature of 800 °C) is also studied for the selective hydrogenation of nitroarenes, which exhibits superior activity and reusability.^[^ ^194^ ^]^ Co SAs/NC‐800 achieve a high yield of aniline (99.8%) in aqueous solution under 5 bar H_2_, with a TOF of 18.2 h^−1^. But Co NPs/NC‐800 only account for a yield of 44.7% under the same condition (Figure 11i). The control experiments using catalysts without Co sites imply that the excellent hydrogenation activity of Co SAs/NC‐800 is largely derived from Co‐N_4_ coordination (Figure 11h). The Bader charge analysis suggests the higher electron density of Co single atoms in the Co SAs/NC‐800 than that of Co SAs/NC‐600. The increase in electron density facilitates the denotation of electrons from Co sites to H_2_ as well as the dissociation of H_2_ molecules.

Moreover, isolated iron supported on ordered mesoporous nitrogen‐doped carbon (Fe_1_/N‐C)^[^ ^216^ ^]^ shows superb activity and tolerance for functional groups in the transfer hydrogenation of nitroarenes over hydrazine hydrate. The catalytic performance of Fe_1_/N‐C is superior to that of Fe NP/N‐C catalyst. On the SACs, nitrobenzene is fully hydrogenated to aniline within 2 h at 60 °C, while Fe NP/N‐C catalyst exhibits the lower selectivity of 88% at 16% conversion. And the TOF of Fe_1_/N‐C is 748 h^−1^, much higher than that of Fe NP/N‐C (75 h^−1^). DFT calculations reveal the difference in the energy barrier in the rate‐determining step. The results indicate the Fe_1_/N‐C SACs will be an inspiring catalyst in transfer hydrogenation compared to Fe NPs. Besides, the hydrogenation of the oxygen in the ‐NO_2_ groups is endothermic. On the basis of the data on transition‐state energies and thermodynamics, the reaction pathway on Fe‐N_4_ sites is more feasible compared to that on Fe NPs and the former exhibits more superior catalytic performance.

As has been discussed above, Pt‐based SACs and non‐noble metals supported on nitrogen‐doped carbon material both exhibit superior activity and selectivity in selective hydrogenation of nitroaromatic hydrocarbons (**Table** **1**). The fabrication of SACs, where the metal species are downsized to the atomic level, prevents the reduction of C=C bonds to the most extent. Besides, when H_2_ is heterolytically cleaved, the produced H^+^/H^−^pairs tend to reduce the polar ‐NO_2_ groups rather than the nonpolar C=C groups.^[^ ^203^ ^]^ The heterolysis of H_2_ is generally considered to occur on the metal‐support interfacial sites,^[^ ^217^ ^]^ which are maximized in SACs. That's why SACs show remarkable selectivity compared to their NP counterparts.

### 3.2 Selective Hydrogenation of Unsaturated Aldehyde

Selective hydrogenation of unsaturated aldehydes (e.g., crotonaldehyde) to unsaturated alcohols represents another group of industrially important reactions to prepare the reaction intermediates for synthesizing perfumes, flavorings, and pharmaceuticals. Notably, the C=C bonds are more sensitive and prone to reduction than the C=O bonds based on both thermodynamic (favored by ca. 35 kJ mol^−1^) and kinetic considerations. Therefore, it is a challenging task to implement such a highly selective and desirable reaction.^[^ ^220^, ^221^, ^222^, ^223^ ^]^

Homogeneous catalysts are efficient for this family of reactions through the formation of ionic hydrogen species, which selectively activates the C=O bonds in *α*, *β*‐unsaturated aldehydes.^[^ ^224^, ^225^, ^226^, ^227^, ^228^ ^]^ However, the homogeneous catalysts are not environmentally friendly, which is also inconvenient to be separated from the products and recycled.^[^ ^229^ ^]^ Therefore, researchers have been committed to designing heterogeneous catalysts,^[^ ^230^, ^231^, ^232^, ^233^, ^234^ ^]^ especially supported metal catalysts targeting at overcoming the thermodynamic constraints.^[^ ^235^ ^]^

**Figure 12:**
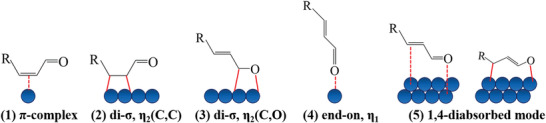
Adsorption patterns of *α*, *β*‐unsaturated aldehydes on metal single atoms and NPs.

*α*, *β*‐unsaturated aldehydes can be adsorbed on the supported metal catalysts via different patterns depending on the configuration of metal species (**Figure** **12**).^[^ ^9^, ^236^, ^237^ ^]^ Obviously, the end‐on mode benefits the preferential hydrogenation of the C=O groups and the desired products of unsaturated alcohols will be obtained.

To facilitate the adsorption of C=O bonds, one of the most effective strategies is to use SACs. These catalysts are reported to display excellent selectivity in the selective hydrogenation of *α*, *β*‐unsaturated aldehydes. Many efforts have been devoted to exploring the catalytic performance of SACs in this family of reactions.

Lou and co‐workers anchor single Rh (Rh_1_) atoms to the edges of MoS_2_ nanosheets to fabricate Rh_1_/MoS_2_ SACs, which can efficiently convert crotonaldehyde toward crotyl alcohol.^[^ ^45^ ^]^ Under moderate conditions of 80 °C and 5 bar H_2_, the Rh_1_/MoS_2_ SAC yields 100% selectivity and TOF of 26.6 h^−1^, while the nano‐Rh/MoS_2_ catalyst yields only 47% selectivity, with 1‐butanol being the main product, and TOF lower than that on Rh_1_/MoS_2_ SAC. Characterization and DFT calculations results both demonstrate that the particular geometric and electronic configuration of Rh_1_/MoS_2_ SACs confines the adsorption mode of crotonaldehyde molecules by a steric effect. The DFT calculations reveal the facile dissociation of H_2_ molecules on the Rh_1_ atoms anchored to the edge of MoS_2_ and the spillover of H atoms to react with the edge O atoms, forming OH moieties and creating a pocketlike HO‐Mo‐Rh_1_‐Mo‐OH configuration. Many parameters of the pocket such as geometric size, configuration, and the electronic structure have a significant influence on the reaction selectivity, similar to the pocket sites of enzymes.^[^ ^238^, ^239^, ^240^ ^]^

**Figure 13:**
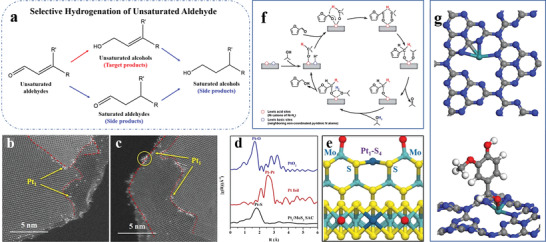
Selective hydrogenation of unsaturated aldehyde. a) Schematic illustration. b–e) The geometric configuration of MoS_2_ edge anchored single Pt atoms. Reproduced with permission.^[^ ^94^ ^]^ Copyright 2021, Wiley‐VCH. f) Possible schematic representation of the mechanism for the catalytic transfer hydrogenation of furfural on Ni‐N_4_ single‐atom sites. Reproduced with permission.^[^ ^218^ ^]^ Copyright 2021,Royal Society of Chemistry. g) Geometries of Ru_1_/mpg‐C_3_N_4_ SACs and adsorption modes. Reproduced with permission.^[^ ^219^ ^]^ Copyright 2018, American Chemical Society.

Similarly, Pt_1_/MoS_2_ SACs (**Figure** **13**b–e) also exhibits excellent intrinsic activity in the selective hydrogenation of crotonaldehyde.^[^ ^94^ ^]^ Under mild conditions of 75 °C and 6 bar H_2_, Pt_1_/MoS_2_ SACs exhibit 100% selectivity toward crotyl alcohol and the TOF is 40.5 h^−1^. However, the activity of nano‐Pt/MoS_2_ catalyst is much lower and the selectivity to crotyl alcohol is only 41%, with the main product 1‐butanol. The unique pocket‐like active sites of Pt_1_/MoS_2_ SACs confine the adsorption configuration of reactant molecules and boost the dissociation of H_2_, thus simultaneously improving the catalytic selectivity and activity. The Pt_1_‐S_4_ coordination accounts for the unique electronic structure of Pt_1_ atoms. H_2_ molecules can be easily dissociated on Pt single‐atom sites and then spill over to produce OH species, forming the HO‐Mo‐S‐Pt_1_‐S‐Mo‐OH configuration, which confines the adsorption modes of crotonaldehyde and achieves high selectivity.

Apart from crotyl alcohol, furfuryl alcohol is also a practical hydrogenation product, which can be obtained from furfural and is widely used in the production of synthetic resins, reactive solvents, and other chemicals.^[^ ^241^, ^242^, ^243^ ^]^ Hu and co‐workers synthesized Pd_1_/C_3_N_4_ SAC by confining the Pd single atoms into the sixfold N‐coordinating cavities of graphitic carbon nitride (g‐C_3_N_4_), which is utilized for the selective hydrogenation of furfural.^[^ ^244^ ^]^ It can be noticed that Pd_1_/C_3_N_4_ delivers 99% selectivity toward furfuryl alcohol and 64% conversion when reacting for 4 h, and it possesses a significantly high activity that the TOF is 146 h^−1^. By contrast, Pd NPs/C_3_N_4_ catalyst exhibits lower activity that the TOF is 67 h^−1^, along with 99% selectivity toward furfuryl alcohol at 39% conversion under the same conditions. In addition, the SACs show excellent durability and thermal stability. The EXAFS fitting results of Pd_1_/C_3_N_4_ show each Pd single atom coordinates with about three N atoms (Pd_1_‐N_3_) on C_3_N_4_ support, implying that the MSI contributes to the distinct coordination environment of single Pd atoms.

The high‐pressure reactions using H_2_ as an H‐donor has many drawbacks, such as high inflammability and instrumental requirements as well as risk of explosion.^[^ ^245^, ^246^ ^]^ Catalytic transfer hydrogenation reaction employs low‐cost and readily available alcohol compounds as the H‐donor, which has received increasing attention and is greener, more effective, and safer.^[^ ^247^ ^]^

Fan's group has explored the employment of SACs for the catalytic transfer hydrogenation of furfural toward furfuryl alcohol (Figure 13f).^[^ ^218^ ^]^ Atomically dispersed Ni‐N_4_ sites in Ni‐SAs/NC change the electron density at the metal center and exhibit unique adsorption and desorption to furfural and furfuryl alcohol, facilitating efficient hydrogen transfer.^[^ ^248^, ^249^ ^]^ The ultra‐high atom utilization efficiency maximizes the number of active sites, improving the reaction efficiency to the highest level. The excellent pore structure enables Ni monatomic active sites to be adequately exposed, and meanwhile, the pore size is favorable to the mass transfer between substrates and products. As a result, the SACs exhibit outstanding catalytic performance that TOF reaches 832 h^−1^ and selectivity is as high as 97.1%, with a conversion level of 85.1% at 130 °C when reacting for 2 h. In contrast, the Ni‐NPs/NC catalysts acquire 57.2% conversion and 95.1% selectivity, along with a TOF value of 69 h^−1^.

Isolated single Ru atom supported on a mesoporous carbon nitride (Ru_1_/mpg‐C_3_N_4_) is explored for another biomass molecule vanillin.^[^ ^219^ ^]^ It's a remarkable fact that selectivity of ≈100% and conversion up to 95% are achieved in the hydrogenation of vanillin to vanillyl alcohol at 60 °C over the SACs. The conversion and selectivity of Ru NPs/mpg‐C_3_N_4_ catalysts are lower than that of Ru_1_/mpg‐C_3_N_4_, which is 29% and 95% respectively. The researchers carry out a systematic study of the reaction mechanism on Ru SACs in the hydrogenation of vanillin, through first‐principles calculations, and propose the dominant structure of vanillin hydrogenation (Figure 13g).

In summary, single transition metal, especially noble metal atoms supported on 2D nanomaterials (e.g., MoS_2_ and g‐C_3_N_4_) with distinctive structural and electronic properties and other appropriate substrates exhibit superior activity and selectivity in selective hydrogenation of unsaturated aldehydes (Table 1). Though the C=C bonds are more prone to reduction, the end‐on adsorption mode of substrates via the O atom on the single‐atom sites in an oxidation state is the key point to achieving high selectivity.

### 3.3 Selective Hydrogenation of Alkyne

Olefins with a low number of carbon atoms (e.g., ethylene, propylene, and butene), are named after light olefins. They are industrially important raw materials for the production of plastics (e.g., polyethylene, polypropylene) and other chemicals.^[^ ^255^, ^256^ ^]^ Take ethylene as an example, it is routinely acquired by steam cracking of petroleum hydrocarbons (e.g., naphtha, gas oil, and condensates). A trace amount of acetylene inevitably exists as a co‐product.^[^ ^257^, ^258^ ^]^ Excess acetylene is poisonous to Ziegler–Natta catalyst in the polymerization of ethylene. Consequently, it is an indispensable step to remove residuary acetylene from ethylene in the production of polyethylene.^[^ ^69^ ^]^

Semi‐hydrogenation of acetylene is a significant and challenging step in the polyethylene industry to remove trace amounts of acetylene effectively and environmental‐friendly. In conventional nanometer‐sized metal particles/clusters, a large proportion of metal atoms are buried in the inner part so that the atomic utilization rate is relatively low^[^ ^259^, ^260^, ^261^ ^]^ while each active metal atom is exposed in the supported SACs, which have been explored for this family of reactions.

#### 3.3.1 Pd‐based SACs

Pd‐based catalysts are so far regarded as one of the most efficient catalysts for the semi‐hydrogenation of alkynes due to their excellent hydrogenation activity^[^ ^262^ ^]^ and researchers have paid much attention to supported nanometer‐sized Pd particles/clusters.^[^ ^261^, ^263^, ^264^ ^]^ Notably, it's beneficial to figure out the influence of the structure of the catalysts on their performance in semi‐hydrogenation reactions when designing novel catalysts that are both active and selective.

**Figure 14:**
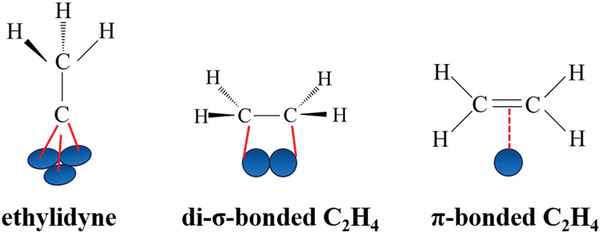
Different adsorption patterns of ethylene on Pd catalysts with different structures.

The ensemble effect plays a primary role in determining the selectivity in the semi‐hydrogenation of alkynes. Ethylene has three adsorption patterns according to different assemblies of Pd species, that is, ethylidyne mode on Pd trimers, di‐*σ*‐mode on bridged Pd dimers, and *π*‐bonded mode on isolated Pd single atoms (**Figure** **14**). The adsorption strength decreases in sequence of ethylidyne > di‐*σ* > *π*‐bonded.^[^ ^8^, ^265^ ^]^ When ethylene is adsorbed through the ethylidyne or di‐*σ*‐mode, the selectivity of semi‐hydrogenation to ethylene is low. At the moment, the deep hydrogenation takes precedence since the energy barrier for desorption is higher than that for hydrogenation. Instead, when ethylene is adsorbed through the *π*‐bonded mode, the selectivity to ethylene is higher than that of other modes. The energy required for desorption is lower than that for further hydrogenation on this mode, so that the ethylene facilely desorb from the supported metal catalyst surface rather than further hydrogenation. It means that the Pd‐based SACs with isolated active sites enable to improve the selectivity toward ethylene.^[^ ^262^, ^266^, ^267^ ^]^

**Figure 15:**
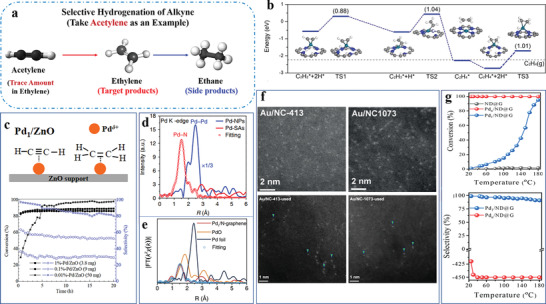
Selective hydrogenation of alkyne. a) Schematic illustration. b) Hydrogenation mechanism of C_2_H_2_ on ISA‐Pd. Reproduced with permission.^[^ ^250^ ^]^ Copyright 2019, Wiley‐VCH. c) Semi‐hydrogenation of C_2_H_2_ over Pd_1_/ZnO. Reproduced with permission.^[^ ^251^ ^]^ Copyright 2016, Elsevier. d) Fourier transforms of k^3^‐weighted Pd K‐edge EXAFS experimental data for Pd‐NPs and Pd‐SAs. Reproduced with permission.^[^ ^252^ ^]^ Copyright 2018, Springer Nature. e) The k^2^‐weighted Fourier transforms of Pd K‐edge EXAFS spectra for Pd_1_/N‐graphene, PdO, and Pd foil. Reproduced with permission.^[^ ^69^ ^]^ Copyright 2021, Wiley‐VCH. f) HAADF‐STEM images of the Au/NC‐413 and Au/NC‐1073 catalysts before and after 2‐methyl‐3‐butyn‐2‐ol semi‐hydrogenation reaction. Reproduced with permission.^[^ ^253^ ^]^ Copyright 2018, Wiley‐VCH. g) Conversion and selectivity for acetylene semi‐hydrogenation over Pd_n_/ND@G and Pd_1_/ND@G catalysts. Reproduced with permission.^[^ ^254^ ^]^ Copyright 2018, American Chemical Society.

Pd‐based catalysts that are fabricated by anchoring the isolated Pd atoms onto the defective nanodiamond‐graphene (Pd_1_/ND@G) are utilized for the selective hydrogenation of acetylene in the presence of abundant ethylene.^[^ ^254^ ^]^ The Pd_1_/ND@G SACs manifest remarkable catalytic activity and ethylene selectivity (>90%), with a 100% conversion at 180 °C. The selectivity of ethylene over Pd_n_/ND@G is ‐450%, demonstrating that abundant ethylene in the feed is transformed to ethane (**Figure** **15**g). The further hydrogenation process yields low‐value products while wasting the raw ethylene. One can conclude from the DFT calculations that, though the fundamental steps for deep hydrogenation of C_2_H_4_ to C_2_H_6_ are still thermodynamically exothermic at Pd single‐atom active sites, the desorption energy of surface C_2_H_4_ species to the gas phase is quite lower than the energy barrier of further hydrogenation of adsorbed C_2_H_4_ intermediate to C_2_H_6_. The competition of ethylene desorption at the active sites of Pd_1_/ND@G accounts for the high selectivity of the semi‐hydrogenation acetylene.

It has been reported that Pd single‐atom active sites are anchored on the inner walls of mesoporous N‐doped carbon foam nanospheres (ISA‐Pd/MPNC).^[^ ^250^ ^]^ Compared to NP‐Pd/MPNC catalyst that favors the formation of undesired ethane, the ISA‐Pd/MPNC catalyst outperforms it in ethylene selectivity (the former is 17%, and the latter is 82%) obviously. The researchers owe the high ethylene selectivity to the feature of single‐atom active sites in the ISA‐Pd/MPNC catalyst. It is generally recognized that only the Pd single‐atom sites exposed on the surface contribute to the catalytic activity, while Pd atoms in the inner part cannot participate in the reaction. All the isolated single Pd atoms can all be completely exposed because of the high specific surface area and thin pore wall. Moreover, the mesoporous foam structure promotes the mass transport. Due to the above two reasons, the SACs possess high activity. Additionally, DFT calculations show ethylene prefers desorption rather than further hydrogenation to C_2_H_6_ (Figure 15b).

Nitrogen‐doped carbon (CN) originating from a MOF material (ZIF‐8) is utilized as the anchoring support to seize the migrating metal atoms that are transformed from NPs at high temperatures to obtain Pd‐SAs (Figure 15d).^[^ ^252^ ^]^ It's inspiring that the thermostable Pd‐SAs show enhanced catalytic performance in the semi‐hydrogenation of acetylene, whose acetylene conversion is up to 96.0% and the selectivity to ethylene is high (93.4%) at 120 °C. By comparison, Pd‐NPs/CN shows worse activity and selectivity. The catalysts only convert 70.1% acetylene and deliver 71.8% selectivity at the same temperature. DFT calculations illustrate the reason for the better catalytic performance of Pd‐SAs than Pd‐NPs. Resembling other catalysts, two barriers are required to be overcome on Pd‐SAs in the hydrogenation of ethylene to ethane and one of the locations of the transition state (0.89 eV) is above the ethylene line. Hence further hydrogenation is prevented at Pd‐SAs sites. But on Pd (111) surface, acetylene and ethylene can be absorbed on several Pd atoms. The further hydrogenation barriers are lower than the chemical adsorption energy of ethylene so that further hydrogenation of ethylene toward ethane is dominant during the whole process on Pd‐NPs and yields a low selectivity of ethylene.

#### 3.3.2 Au‐based SACs

The selective hydrogenation of alkynes on Au‐based catalysts is normally attributed to thermodynamic factors. DFT simulations suggest that only the C≡C bonds are adsorbed and activated on Au catalysts, while the adsorption strength of C=C bonds is too weak to be activated. Thus, molecules containing C=C bonds readily leave and only C≡C bonds can be hydrogenated on Au catalysts, resulting in the selective hydrogenation of alkynes even in the presence of abundant alkenes.^[^ ^268^ ^]^ Furthermore, it is relatively difficult to dissociate H_2_ on Au catalysts that enable to constrain the over‐hydrogenation reactions.^[^ ^269^, ^270^ ^]^

It has been discovered that the isolated Au^3+^ ions distributed on the ZrO_2_ surface possess two orders of magnitude higher mass‐specific activity than that of Au NPs for the selective hydrogenation of 1, 3‐butadiene to butenes.^[^ ^31^ ^]^ Encouragingly, all the tested Au/ZrO_2_ catalysts exhibit 100% selectivity for butene in the hydrogenation of 1, 3‐butadiene. Though 0.08% Au/ZrO_2_ catalysts are the most active, no butane is detected when the conversion of 1, 3‐butadiene is high. The catalytic activity over Au/ZrO_2_ catalysts with higher Au mass loading is sufficiently lower, which implies that the highly active Au^3+^ ions are those independently anchored at specific sites on the surface of ZrO_2_.

Furthermore, the influences of Au^3+^/Au^0^ ratio or distribution of Au oxidation states in Au/ZrO_2_ catalysts with various Au loading (0.01–0.76 wt.%) on the hydrogenation of 1,3‐butadiene are investigated by changing the temperature of catalyst calcination and prereduction with H_2_.^[^ ^271^ ^]^ TPR (Temperature Programmed Reduction) results demonstrate that all Au atoms in Au/ZrO_2_ samples (only 0.08 wt.% Au) are in the form of reducible Au^3+^ ions. 0.05% Au/ZrO_2_‐473 and 0.08% Au/ZrO_2_‐473 catalysts yield 100% selectivity for butene, among which 60% is the more valuable 1‐butene. There are only isolated Au^3+^ ions in the above two catalysts. In contrast, butane will be formed over 0.76% Au/ZrO_2_‐473 catalysts, where metallic Au particles exist.

A MOF containing an Au (III) Schiff base complex lining the pore walls (IRMOF‐3‐SI‐Au) is reported for the selective hydrogenation of 1,3‐butadiene.^[^ ^272^ ^]^ The cationic Au(III) species are strongly stabilized and the utilization rate of Au active sites reaches 100%, which displays excellent catalytic performance. The selectivity for butene is up to 97% at almost complete consumption of 1, 3‐butadiene (96%) with TOF of 540 h^−1^ and the main products are 1‐butene and E‐2‐butene.

To summarize, the Pd‐based and Au‐based SACs exhibit superior activity and selectivity in selective hydrogenation of alkyne (Table 1). Based on the theoretical understanding of the adsorption behaviors of alkyne/alkene on metal surfaces, the adsorption strength of the alkenes over homogeneous active sites of those two types of SACs is weak and alkenes tend to desorb from the catalyst surface, consequently enhancing the chemoselectivity.

### 3.4 Selective Hydrogenation of CO_2_

Since greenhouse gases (mainly CO_2_) have brought out a series of environmental problems, it is urgent to reduce these gases in the atmosphere. Hydrogenation of CO_2_ represents a group of industrially valuable transformations. Not only does it facilitate the alleviation of the greenhouse effect caused by the increase of CO_2_ content in the atmosphere, but also provides high‐value chemicals and reduces our dependence on fossil fuels, consistent with the “carbon neutral” economy.^[^ ^276^, ^277^, ^278^, ^279^, ^280^, ^281^ ^]^ Multiple reaction paths unavoidably coexist over practical catalysts so that various products (e.g., CO, CH_4_, HCOOH, CH_3_OH, higher alcohols, and even gasoline) may be generated, influencing the selectivity of the target product.^[^ ^282^, ^283^, ^284^ ^]^ It's highly desired to develop catalysts with high activity and selectivity for CO_2_ hydrogenation, especially efficiently controlling the reaction path.

For CO_2_ hydrogenation into methanol, Pt_1_@MIL catalysts improve the selectivity by inducing a distinct reaction path. The active sites consist of a Pt single atom and its coordinated oxygen atoms in MIL‐101.^[^ ^285^ ^]^ According to mechanistic studies, H_2_ is first dissociated on Pt species to form Pt‐H bonds. Afterward, a Pt single atom activates its coordinated O atoms to adsorb the H atoms and two ‐OH groups are formed, wherein the H atom of a hydroxy group is added into CO_2_ to produce HCOO* intermediates. It's generally reported that HCOO* will be hydrogenated into HCOOH* and eventually converted to methanol.^[^ ^286^, ^287^ ^]^ With regard to Pt_n_@MIL, H_2_ is directly dissociated on Pt NPs to generate Pt‐H species. CO_2_ is hydrogenated into COOH* intermediates by utilizing the H atoms offered by Pt hydrides. COOH* easily loses the hydroxyl species to form CO* species.^[^ ^286^, ^288^ ^]^ CO* can be either desorbed to form CO in the gas phase or further hydrogenated to CH_4_.^[^ ^286^ ^]^ Hence the adsorption features of H atoms on Pt_1_@MIL and Pt_n_@MIL induce different reaction paths in CO_2_ hydrogenation. Not only does the unique reaction path on Pt_1_@MIL reduce the activation energy to enhance the catalytic activity, but also accounts for the high selectivity of methanol. As a result, under conditions of 32 bar and 150 °C, the SACs achieve the TOF of 117 h^−1^ in DMF, 5.6 times as high as that of Pt_n_@MIL. The selectivity of methanol over Pt_1_@MIL reaches 90.3%. However, the selectivity for methanol over Pt_n_@MIL is only 13.3% and the major product is CO.

**Figure 16:**
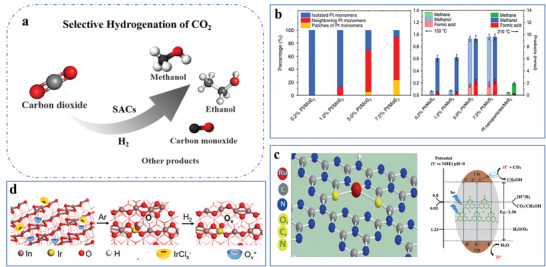
CO_2_ hydrogenation to various products. a) Schematic illustration. b) Histogram of the contents of isolated, neighboring, and patches of Pt monomers in Pt/MoS_2_ SACs with different Pt loadings and catalytic results of Pt/MoS_2_ catalysts for the hydrogenation of CO_2._ Reproduced with permission.^[^ ^273^ ^]^ Copyright 2018, Springer Nature. c) Ru coordinating with N in the CN matrix of RuSA‐mC_3_N_4_ photocatalyst and proposed photocatalysis mechanism over its surface. Reproduced with permission.^[^ ^274^ ^]^ Copyright 2021, Wiley‐VCH. d) Schematic illustration of the fabrication procedures of Ir_1_‐In_2_O_3_. Reproduced with permission.^[^ ^275^ ^]^ Copyright 2020, American Chemical Society.

Under ambient environment, porous carbon nitride supported Ru single atoms (RuSA‐mC_3_N_4_) as photoactive catalysts enable the conversion of CO_2_ toward methanol with a yield of 1500 µmol g^−1^ in 6 h under visible light, higher than that of nano Ru‐gC_3_N_4_ and graphitic Ru‐C_3_N_4_.^[^ ^274^ ^]^ The possible reasons for the higher photocatalytic activity for RuSA‐mC_3_N_4_ are lower bandgap, the prolonged average lifetime of photogenerated electron‐hole pair, and facile electron transfer between metal and semiconductor (**Figure** **16**c).

Generally speaking, to broaden the light response, the bandgap is narrowed to generate more electrons upon irradiation. Compared to CN, the adsorption in the visible range over RuSA‐mC_3_N_4_ is enhanced, suggesting Ru doping narrows the bandgap and thus improves the light‐harvesting ability in the visible‐light range. After evaluating the recombination rate of electron‐hole pairs, it is concluded that charge carrier recombination is inhibited and can survive longer over RuSA‐mC_3_N_4_. Moreover, according to characterization analyses, Ru is bound to sites through Ru—C/N (and/or Ru‐O). These sites may perform as a bridge, which contributes to the faster electron transfer and enhance the charge density on Ru. Therefore, the photocarrier transfer barrier is reduced and the photocatalytic activity of RuSA‐mC_3_N_4_ for the aqueous reduction of CO_2_ to methanol is augmented.

The metal loading on the above two catalysts is kept low (<1.0%) so that the single atoms are far apart and the interaction between the single atoms can be generally negligible. Pt/MoS_2_ catalysts with the Pt loading as high as 7.5% while still remaining the atomic dispersion of Pt species are efficient for conversion into methanol.^[^ ^273^ ^]^ Each active center consists of a Pt atom and its corresponding activated S atoms. While increasing the Pt loading on MoS_2_, the distance between the active centers become smaller. If two active centers are partly overlapped or adjacent, the two related Pt atoms are considered as neighboring Pt monomers.

The synergistic effect between neighboring Pt monomers over MoS_2_ apparently enhances the catalytic activity for the hydrogenation of CO_2_ and reduces the activation energy compared to isolated Pt monomers. It's revealed from the mechanistic studies that CO_2_ is transformed into methanol without forming HCOOH intermediates over isolated Pt monomers. However, neighboring Pt monomers act synergistically to change the energy barriers and reaction pathways, where CO_2_ is converted into HCOOH and CH_3_OH successively. CH_2_OH* is the main intermediate on 0.2% Pt/MoS_2_ while COOH* is dominant on 7.5% Pt/MoS_2_. As a result, when the Pt loading is increased from 0.2% to 7.5%, the selectivity to methanol decreases from 95.4% to 81.3% at 150 °C and from 93.0% to 76.0% at 210 °C after 3 h. As a control, 0.041 mmol of methane and 0.002 mmol of formic acid are produced over MoS_2_‐supported Pt NPs at 150 °C after 3 h. And when performed at 210 °C, the total production of products is 2.0 mmol and majority (87.2%) of products is still methane (Figure 16b). It's obvious that the performance of Pt NPs is poorer than the atomically dispersed Pt/MoS_2_.

In terms of the conversion of CO_2_ to ethanol, Ir_1_‐In_2_O_3_ SAC yields a high selectivity of >99% along with the initial TOF of 481 h^−1^. The yield of ethanol and value of TOF decreases with the increase of Ir loadings.^[^ ^275^ ^]^ Besides the partially reduced In_2_O_3_, the Ir_1_ atoms and neighboring oxygen vacancy (O_v_) generate a Lewis acid‐base pair, which are both important compositions of Ir_1_‐In_2_O_3_ SAC. The catalysts facilitate the adsorption and activation of CO_2_ to CO*. Apart from that, they make a positive contribution to C—C coupling between CH_3_O*‐O_v_ and Ir^*δ*+^‐CO* (Figure 16d).

CeO_2_‐supported Pd dimers (Pd_2_/CeO_2_) are reported to possess brilliant catalytic performance in the conversion of CO_2_ to ethanol.^[^ ^276^ ^]^ The specific Pd_2_O_4_ configuration and high uniformity of Pd dimers make it easy for CO_2_ to be activated. CO_2_ is directly dissociated to CO intermediate, and C‐C coupling is triggered. In the meantime, further C_2+_ coupling is inhibited, thus leading to the high selectivity to ethanol. Besides, the significant charge transfer between CO and Pd species enables CO to strongly bind via the Pd_2_O_4_ configuration on Pd_2_/CeO_2_, which prohibits CO desorption and facilitates the coupling between CO and CH_3_ intermediates, leading to the formation of the precursor of ethanol. The conversion of CO_2_ is 9.2%, higher than that of nano‐Pd/CeO_2_ catalyst. Moreover, the Pd_2_/CeO_2_ catalyst gives an enhanced selectivity of 99.2% toward ethanol and the space‐time yield is 45.6 g_ethanol_ g_Pd_^−1^ h^−1^ while nano‐Pd/CeO_2_ catalyst largely forms CO.

In regard to reverse water gas shift reaction (RWGS, CO_2_ + 3H_2_ ↔CO+2H_2_ + H_2_O), Pd single atoms dispersed on *α*‐MoC with up to 5 wt.% Pd loading are studied and found to deliver high activity and selectivity.^[^ ^289^ ^]^ Since the formation energy of Mo‐vacancy on the *α*‐MoC surface is much lower than that of C‐vacancy, it is thermodynamically favorable to form the Mo‐vacancy. And the theoretical results convincingly reveal that Pd single atoms are stabilized at the Mo vacancy of the *α*‐MoC substrate surface through orbital interaction as well as charge transfer between Pd atoms and the neighboring C atoms. Under conditions of 400 °C in CO_2_:H_2_ = 1:3, the selectivity to CO on SACs reaches 98.2%. Pd NP/*α*‐MoC‐EG (EG: Ethylene Glycol) catalyst is also tested as a control, and the CO selectivity is much lower than Pd_1_/*α*‐MoC SACs catalyst.

As what has been discussed on the selective hydrogenation of CO_2_, SACs, especially noble metal‐based SACs, play an important role in transforming CO_2_ into valuable chemicals. Apart from improving the atomic utilization efficiency, dispersed monatomic active sites alter the reaction path by influencing the adsorption patterns of reactants or intermediates on catalysts, thus leading to high selectivity to desired products (Table 1).

**Table 1:** Comparison of SACs and Nano Catalysts for Selective Hydrogenation

Selective hydrogenation of nitroaromatic hydrocarbons							
Substrate	Catalyst	*T* [°C]	*P* [MPa]	Conv. [%]	Sel. [%]	TOF [h^−1^]	Ref
3‐nitrostyrene	0.08%Pt/FeO_x_‐R250^a)^	40	0.3	96.5	98.6	1514	[148]
3‐nitrostyrene	4.30%Pt/FeO_x_‐R250^b)^	40	0.3	94.2	92.7	762	
3‐nitrostyrene	Pt_1_/Ni nanocrystals^a)^	40	0.3	>99	>99	1800	[26]
3‐nitrostyrene	Pt clusters/Ni nanocrystals^b)^	40	0.3	>99	19	865	
nitrobenzene	Co SAs/NC^a)^	110	3	99.7	99.1	76.8	[213]
nitrobenzene	Co NPs/NC^b)^	110	3	84.8	97.4	14.3	
nitrobenzene	Co SAs/NC‐800^a)^	120	0.5	100	99.8	18.2	[194]
nitrobenzene	Co NPs/NC‐800^b)^	120	0.5	45.2	98.9	8.2	
nitrobenzene	Fe_1_/N—C^a)^	60	/	99	99	748	[216]
nitrobenzene	Fe NP/N—C^b)^	60	/	16	88	75	

Selective hydrogenation of unsaturated aldehyde							
Substrate	Catalyst	*T* [°C]	*P* [MPa]	Conv. [%]	Sel. [%]	TOF [h^−1^]	Ref
crotonaldehyde	Rh_1_/MoS_2_ ^a)^	80	0.5	/	100	26.6	[45]
crotonaldehyde	nano‐Rh/MoS_2_ ^b)^	80	0.5	/	47	<5	
crotonaldehyde	Pt_1_/MoS_2_ ^a)^	75	0.6	/	100	40.5	[94]
crotonaldehyde	nano‐Pt/MoS_2_ ^b)^	75	0.6	/	42.6	9.9	
furfural	Pd_1_/C_3_N_4_ ^a)^	90	0.1	64	99	146	[244]
furfural	Pd NPs/C_3_N_4_ ^b)^	90	0.1	39	99	67	
furfural	Ni‐SAs/NC^a)^	130	2	85.1	97.1	832	[218]
furfural	Ni‐NPs/NC^b)^	130	2	57.2	95.1	69	
vanillin	Ru_1_/mpg‐C_3_N_4_ ^a)^	60	4	95	≈100	/	[219]
vanillin	Ru NPs/mpg‐C_3_N_4_ ^b)^	60	4	29	95	/	

Selective hydrogenation of alkyne (take acetylene as example)							
Catalyst	Composition	*T* [°C]	*P* [MPa]	Conv. [%]	Sel. [%]	SV [mL g_cat_^−1^·h^−1^]	Ref
Pd_1_/ND@G^a)^	C_2_H_2_/C_2_H_4_/H_2_ = 1/20/10	180	0.1	100	90	60 000	[254]
Pd_n_/ND@G^b)^	C_2_H_2_/C_2_H_4_/H_2_ = 1/20/10	180	0.1	100	‐450%		
Pd_1_/N‐graphene^a)^	C_2_H_2_/C_2_H_4_/H_2_ = 1/20/20	125	0.1	99	93.5	1200	[69]
Pd NPs/N‐graphene^b)^	C_2_H_2_/C_2_H_4_/H_2_ = 1/20/20	125	0.1	52	53		
0.01%Pd_1_/ZnO^a)^	C_2_H_2_/C_2_H_4_/H_2_ = 2/20/40	80	0.1	100	>80	36 000	[251]
1%Pd_1_/ZnO^b)^	C_2_H_2_/C_2_H_4_/H_2_ = 2/20/40	80	0.1	≈90	≈30	473 684	
ISA‐Pd/MPNC^a)^	C_2_H_2_/C_2_H_4_/H_2_ = 0.5/50/5	110	0.1	83	82	10 500	[250]
NP‐Pd/MPNC^b)^	C_2_H_2_/C_2_H_4_/H_2_ = 0.5/50/5	110	0.1	100	17	210 000	
Pd‐SAs/CN^a)^	C_2_H_2_/C_2_H_4_/H_2_ = 0.5/50/5	120	0.1	96	93.4	/	[252]
Pd‐NPs/CN^b)^	C_2_H_2_/C_2_H_4_/H_2_ = 0.5/50/5	120	0.1	70.1	71.8	/	

Selective hydrogenation of CO_2_							
Catalyst	Major products	*T* [°C]	*P* [MPa]	Sel. [%]	TOF [h^−1^]	Yield	Ref
Pt_1_@MIL^a)^	CH_3_OH	150	3.2	90.3	117	1.2 mmol g^−1^ h^−1^	[285]
Pt_n_@MIL^a)^	CH_3_OH	150	3.2	13.3	20.9	0.5 mmol g^−1^ h^−1^	
7.5%Pt/MoS_2_ ^a)^	CH_3_OH	210	3.2	76	308.8	8 mol g_Pt_^−1^ h^−1^	[273]
Pt nanoparticles/MoS_2_ ^b)^	CH_4_	210	3.2	87.2	/	1.7 mol g_Pt_^−1^ h^−1^	
Ir_1_‐In_2_O_3_ ^a)^	C_2_H_5_OH	200	6	>99	481	0.99 mmol g^−1^ h^−1^	[275]
1 wt%Ir_1_‐In_2_O_3_ ^b)^	CH_3_OH	200	6	94	≈0	/	
Pd_2_/CeO_2_ ^a)^	C_2_H_5_OH	240	3	99.2	211.7	45.6 g g_Pd_^−1^ h^−1^	[276]
nano‐Pd/CeO_2_ ^b)^	CH_3_OH	240	3	27.0	26.5	0.5 g g_Pd_^−1^ h^−1^	

a) SACs

^b)^ Nano catalysts

## 4 Selective Oxidation over SACs

### 4.1 Selective Oxidation of Methane to C1 Oxygenates

Methane is one of the most abundant feedstocks for chemical production, which can be extracted from natural gas, shale gas, and methane hydrate.^[^ ^290^, ^291^, ^292^, ^293^ ^]^ Unfortunately, as methane is a potent greenhouse gas,^[^ ^294^, ^295^ ^]^ its emission and combustion could exacerbate global warming. Therefore, efficiently converting methane to value‐added chemicals is of great significance.^[^ ^296^ ^]^

The first C‐H bond in the methane molecule has extremely high bond energy (439.3 kJ mol^−1^),^[^ ^297^, ^298^ ^]^ which makes its cleavage (homolysis or heterolysis) very difficult. Currently, the indirect methane converting pathways include steam reforming of methane (SRM) and dry reforming of methane (DRM), in which thermal energy provides a large amount of energy to split C‐H bonds, resulting in high energy costs.^[^ ^296^, ^299^, ^300^, ^301^, ^302^, ^303^ ^]^ Meanwhile, the target product is prone to further over‐oxidation to CO_2_, resulting in low selectivity. Converting methane by direct routes has been investigated from the aspects of enzyme catalysis,^[^ ^304^, ^305^, ^306^, ^307^, ^308^, ^309^ ^]^ homogeneous catalysis,^[^ ^310^, ^311^, ^312^ ^]^ and heterogeneous catalysis.^[^ ^313^ ^]^ Nevertheless, direct oxidation of methane to C1 oxygenates in mild conditions with high selectivity remains a huge challenge. As SACs combine the advantages of homogeneous and heterogeneous catalysts,^[^ ^66^ ^]^ researchers have applied them to the direct and selective oxidation of methane and achieved exciting results.

#### 4.1.1 Selective Oxidation of Methane to C1 Oxygenates on Noble Metals

Noble metals like Pd, Pt, Rh, Ir, Ru, and so on have been used to control vehicle emissions, produce chemicals, refine petroleum and serve in fuel cells.^[^ ^66^, ^316^, ^317^ ^]^ As these metals represent the rarest elements on the earth, the efficient utilization of atoms is especially important.^[^ ^318^ ^]^ Although Au—Pd colloid NPs have increased the methanol selectivity to 92%,^[^ ^319^, ^320^, ^321^ ^]^ the metal atom utilization is still not satisfying. Therefore, SACs of noble metals with good homogeneity have been developed for selective oxidation of methane to methanol in recent years.

**Figure 17:**
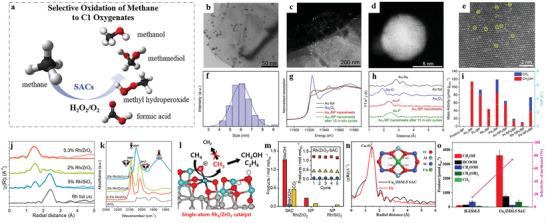
Selective oxidation of methane to C1 oxygenates. a) Schematic illustration of selective oxidation of methane to C1 oxygenates. b–i) Comparison of Au_1_/BP nanosheets and Au NPs/BP nanosheets for selective oxidation of methane. (b) TEM image of Au NPs/BP nanosheets. (c) HAADF‐STEM image of Au NPs/BP nanosheets. (d) HAADF‐STEM image of an individual Au NP. (e) HAADF‐STEM image of Au_1_/BP nanosheets after 10 in situ cycles. (f) Size distribution graph of Au NPs/BP nanosheets. (g) XANES spectrum and (h) EXAFS spectrum of Au_1_/BP nanosheets after 10 in situ cycles. Au foil, Au_2_O_3_, and fresh Au_1_/BP nanosheets were used as the references. (i) Comparison of the catalytic performance of different BP nanosheets‐based catalysts. Reproduced with permission.^[^ ^314^ ^]^ Copyright 2021, Springer Nature. j–m) Rh_1_/ZrO_2_ catalyst for selective oxidation of methane. (j) Rh K edge k^3^‐weighted Fourier transformed EXAFS spectra of Rh/ZrO_2_ SAC (0.3 wt.%), Rh NPs/ZrO_2_ (2 wt.%), Rh/SiO_2_ (5 wt.%), and Rh foil. (k) DRIFT spectra of CO molecules adsorbed on bare ZrO_2_ support, Rh/ZrO_2_ SAC (0.3 wt.%), Rh NPs/ZrO_2_ (2 wt.%), and Rh/SiO_2_ (5 wt.%). (l) Scheme of methane selective oxidation over Rh_1_/ZrO_2_. Reproduced with permission.^[^ ^315^ ^]^ Copyright 2017, American Chemical Society. (m) Catalytic performance of Rh/ZrO_2_ SAC (0.3 wt.%), Rh NPs/ZrO_2_ (2 wt.%), Rh/SiO_2_ (5 wt.%) in methane selective oxidation with H_2_O_2_. The inset shows stability of the Rh/ZrO_2_ SAC. Reproduced with permission.^[^ ^15^ ^]^ Copyright 2020, American Chemical Society. n) EXAFS fitting curve for Cu_1_/ZSM‐5 SAC. Inset, proposed coordination environment of Cu_1_‐O_4_ entity. o) Catalytic performance of Cu_1_/ZSM‐5 SAC and pure H‐ZSM‐5 for direct methane oxidation at 50 °C. Reproduced with permission.^[^ ^48^ ^]^ Copyright 2021, Elsevier.^[^ ^48^ ^]^

Rh is probably one of the most efficient noble metals to catalyze selective oxidation of methane to methanol. Isolated Rh atoms are reported to be dispersed on ZrO_2_ to directly oxidize methane to methanol under mild conditions with hydrogen peroxide.^[^ ^315^ ^]^ According to EXAFS data in **Figure** **17**j, Rh_1_/ZrO_2_ SAC has a dominant peak of Rh‐O at 1.5 Å without an Rh‐Rh peak at 2.4 Å, while Rh NPs/ZrO_2_ has a strong Rh‐O peak with small Rh‐Cl and Rh‐Rh peaks located at 2.0 and 2.4 Å. XANES data indicates that single Rh atoms have a more oxidative state. It is implied that the methyl radical (CH_3_⋅) on the isolated Rh site has the lowest energy during the methane oxidation, helping the dissociative adsorption of methane. CeO_2_ nanowires (NWs) have also been used as a kind of support to stabilize Rh single atoms.^[^ ^46^ ^]^ Compared to Rh clusters supported on CeO_2_, single Rh atoms exhibit the C1 oxygenates yield of 6.5 times higher (1231.7 vs 189.4 mmol g_Rh_^−1^∙h^−1^) with the selectivity of CH_3_OH and CH_3_OOH kept at ≈94%. In situ characterizations and theoretical calculations demonstrate that CeO_2_ NWs facilitate the formation of ∙OOH and ∙OH radicals, while Rh_1_/CeO_2_‐NWs SAC can selectively activate CH_4_ to ∙CH_3_, which can further interact with ∙OOH and ∙OH radicals to produce CH_3_OH and CH_3_OOH.

Moreover, it has been found that the products can be tuned by functionalizing the formed Rh‐CH_3_ via two separate reaction routes: oxygen insertion to form methanol, or CO insertion to form acetic acid.^[^ ^322^ ^]^ Using either zeolite or TiO_2_ as the support of Rh SACs, the methanol yield achieves about 230 mmol g_cat_^−1^, with selectivity up to 100%, when reactions were conducted at 150 °C for 3h. It is believed that the single Rh^+^ cations promote to activate methane by O_2_ to form Rh‐CH_3_ species, which are further converted to Rh‐OCH_3_ species by oxygen insertion with CO ligands existing. The formed Rh‐OCH_3_ can then be hydrolyzed to produce methanol. Nevertheless, the exact mechanism is not clear and requires further investigation. It has also been proposed that the first C‐H bond of CH_4_ can be activated by the Rh single atoms anchored in the ZSM‐5 molecular sieve channel.^[^ ^323^ ^]^ The formed methyl radical couples with CO and OH on the uniform sites of Rh_1_O_5_ and produces acetic acid over a low activation barrier.

Pd SACs also have special homogeneity to boost the direct oxidation of methane to methanol. Atomically dispersed Pd atoms anchored on the inner surface of ZSM‐5 micropores, forming the high uniformity of the Pd_1_O_4_ active site, exhibit excellent methane activation properties.^[^ ^324^ ^]^ With the help of CuO which catalyzes the decomposition of H_2_O_2_ to H_2_O and O_2_, the selectivity for methanol reaches around 86%, significantly higher than that of reactions on PdO NPs. The EXAFS spectrum of Pd foil shows a Pd‐Pd peak at 2.58 Å, while no such peak is observed in the EXAFS spectrum of 0.04 wt.% Pd/ZSM‐5.

Au is another noble metal species that has been reported active in the selective oxidation of methane to methanol. Luo and co‐workers^[^ ^314^ ^]^ report that single Au atoms supported on black phosphorus nanosheets (Au_1_/BP nanosheets) reach >99% selectivity for oxidation of methane to methanol in water under light irradiation. The methanol yield reaches 113.5 µmol g_cat_^−1^ in water under the standard condition of 33 bar of mixed gas (CH_4_:O_2_ = 10:1) with light irradiation (1.2 W, 3.14 cm2) at 90 °C, while the activation energy is 43.4 kJ mol^−1^. According to DRIFTs data, only the peaks for the linear adsorption of CO can be observed for M_1_/BP nanosheets (M = Pt, Pd, and Rh), while the peaks for the bridge adsorption and the linear adsorption can both be observed for M NPs/BP nanosheets. Under the standard condition, the yield of methanol over Au_1_/BP nanosheets is 113.5 µmol g_cat_^−1^, whereas TOF number is 5.6 h^−1^. Remarkably, the TOF number of Au_1_/BP nanosheets is higher than those of Au NPs/BP, M_1_/BP, and M NPs/BP (M = Pt, Rh, and Pd) nanosheets (Figure 17i).

As discussed above, atomically dispersed noble metal atoms behave distinctly differently from the corresponding NPs in the selective oxidation of methane (**Table** **2**). Single atoms anchored on supports can stabilize ·CH_3_ radicals and the MSI^[^ ^90^ ^]^ regulates the selectivity of the oxidation reaction. Up to now, metal oxides and zeolites are the preferred choice of host for SACs of noble metals. Nonetheless, many photosensitive hosts that are beneficial for reactions under mild conditions remain unexplored and their variety of applications is still rather rare.

#### 4.1.2 Selective Oxidation of Methane to C1 Oxygenates on Non‐Noble Metals

For highly efficient CH_4_ oxidation, noble metals are frequently employed as catalysts, which are expensive and rare.^[^ ^310^, ^314^, ^315^, ^319^, ^320^ ^]^ As a result, the application and commercialization of these catalysts have been hindered.^[^ ^325^, ^326^ ^]^ Moreover, studies on methane monooxygenase (MMO) indicate that Fe‐oxygen or Cu‐oxygen structure is the active center of methane conversion, inspiring heterogeneous catalysts to choose zeolites as the supports and Fe and/or Cu as the metal species as an imitation of the structure of MMO.^[^ ^305^, ^306^, ^307^, ^308^, ^309^, ^327^ ^]^ Thus, attempts have been made to catalyze methane on SACs of non‐noble metals.

Single Fe atoms embedded in graphene first exhibit a certain activity towards the direct conversion of methane to C1 oxygenates at room temperature (RT).^[^ ^328^ ^]^ Four carbon atoms are considered to be substituted by four nitrogen atoms in the graphene matrix to form a FeN_4_ structure, which can adsorb two O atoms at each side to form a unique O‐FeN_4_‐O site. The C‐H bond of methane can be activated on the O‐FeN_4_‐O site, and the generated ·CH_3_ is first converted into CH_3_OH or CH_3_OOH, after which CH_3_OH can be further catalyzed to produce HOCH_2_OOH and HCOOH. Atomically dispersed Fe sites confined in ZSM‐5 show excellent efficiency and selectivity in the direct conversion of methane to formic acid.^[^ ^329^ ^]^ The yield of formic acid can reach 383.2 mmol g_cat_^−1^ at 80 °C, with a selectivity of 91%. For producing liquid C1 oxygenates, the turnover frequency achieves 84 200 h^−1^ and outperforms that of Fe particles dispersed in ZSM‐5 that have been reported in the open literature.^[^ ^330^ ^]^ EPR data and DFT calculations prove that the C‐H bonds can be facilely split over the active oxygen species generated on both mononuclear and binuclear Fe sites. With the successive dissociation of C‐H bonds, ·CH_3_, CH_3_OH, ·CH_2_OH, and HCHO are generated to finally produce HCOOH. This research pushes the progress of designing the microenvironment of uniform Fe sites in support of nanochannels to selectively oxidize methane with low energy consumption.

It has already aroused much attention that the uniformity of Cu species in zeolite can improve the activity towards methane direct oxidation, yet the improvement of activity is limited due to the nonuniformity of Cu NPs.^[^ ^331^ ^]^ It has been reported that Cu single atoms loaded on ZSM‐5 are combined with four O atoms to form a highly uniform Cu_1_‐O_4_ entity, which can preferentially activate CH_4_ and prevent the peroxide of CH_3_OH (Figure 17n,o).^[^ ^48^ ^]^ At the reaction time of 30 min and the temperature of 50 °C, the yield of C1 oxygenates can achieve 4800 µmol g_cat_^−1^ with high selectivity of 99%. When the reaction temperature rises to 70 °C, the yield of C1 oxide reaches 12 000 µmol g_cat_^−1^, which is comparable to the best precious metal catalyst. However, Cu NPs exhibit low activity and provide no promotion to the selectivity of methanol. As is illustrated by DFT calculations, ·OH can abstract the hydrogen of OH adsorbed at Cu_1_O_4_ site and form desorbed H_2_O with CH_3_O, which blocks the oxidative dehydrogenation of CH_3_OH by ·OH. Moreover, at Cu_1_‐O_4_ entity, the C‐H bond activation of CH_4_ is found to be even easier than that of CH_3_OH in the aqueous solution, which suppresses the further oxidation of CH_3_OH.

In addition, Cr_1_/TiO_2_ SAC has been used to catalyze the direct oxidation of methane to produce C1 oxygenates with H_2_O_2_ as oxidant under mild conditions.^[^ ^332^ ^]^ A series of Cr_1_/TiO_2_ SACs with Cr content of 0.25 to 8 wt.% are prepared by the wet impregnation method, and the catalysts with the loading of less than 1 wt.% reach the highest yield of 57.9 mol mol_Cr_^−1^. At 1h, the selectivity of obtained C1 oxygenates can achieve nearly 100%, and after 20h reaction at 50 °C, the selectivity is around 93% while the yield of C1 oxygenates reaches 57.9 mol·mol_Cr_^−1^. EPR experiments indicate that single Cr atoms promote the formation of ·CH_3_ and ·OH, and the radicals can react to form CH_3_OH. Besides, ·CH_3_ can interact with ‐OOH on the surface of TiO_2_ to form CH_3_OOH and the formed CH_3_OH can be further oxidized to HOCH_2_OOH and HCOOH. The mechanism is similar to that of the graphene‐confined isolated Fe atoms as discussed above.^[^ ^328^ ^]^

From the above discussions, it can be concluded that catalysts of monoatomic non‐noble metals play an essential role in activating the C‐H bond of CH_4_ and oxidant to form ·CH_3_ and active oxygenated species, which can then react to produce C1 oxygenates.^[^ ^332^ ^]^ The researches on non‐noble metals provide approaches for direct conversion of methane with high efficiency and low costs (Table 2).

### 4.2 Selective Oxidation of Alcohol to Aldehyde

The oxidation of alcohols to aldehydes is a fundamentally important transformation in both organic synthesis and industrial applications.^[^ ^333^, ^334^, ^335^, ^336^, ^337^ ^]^ Aldehydes are valuable intermediates and ingredients for the production of commodity chemicals.^[^ ^338^, ^339^ ^]^ Vapor phase oxidation of benzyl alcohol to benzaldehyde has been broadly researched in the past for its practical significance in producing chlorine‐free benzaldehyde applied in perfumery and pharmaceutical industries.^[^ ^340^, ^341^, ^342^, ^343^, ^344^ ^]^ However, it is very challenging to avoid the total oxidation of benzyl alcohol to carbon oxides in the oxidation process of the vapor phase. As the catalytic efficiency of benzyl alcohol oxidation is relatively low over some conventional supported metal catalysts, SACs have been explored to catalyze the selective oxidation of benzyl alcohol.

**Figure 18:**
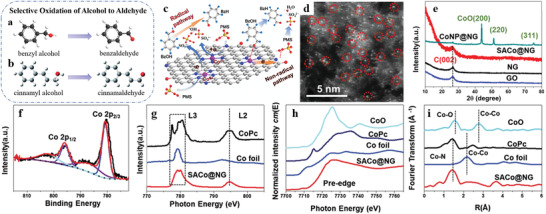
Selective oxidation of alcohol to aldehyde. a) Selective oxidation of benzyl alcohol to benzyl aldehyde. b) Selective oxidation of cinnamyl alcohol to cinnamaldehyde. Reproduced with permission.^[^ ^24^ ^]^ Copyright 2007, Wiley‐VCH. c–i) Selective oxidation of benzyl alcohol on Co_1_@NG. (c) Schematic illustration of the mechanism of Co_1_@NG catalyzing PMS activation and benzyl alcohol oxidation. (d) AC‐STEM‐annular dark‐field (ADF) image of Co_1_@NG. (e) XRD patterns of GO, NG, Co NP@NG, and Co_1_@NG. (f) XPS spectrum of the Co_1_@NG catalyst. (g) Co L‐edge of the NEXAFS spectra of Co_1_@NG, Co foil, and CoPc. (h) XANES Co‐edge and (i) Fourier transform of the EXAFS spectra of Co_1_@NG, Co foil, CoPc, and CoO. Reproduced with permission.^[^ ^346^ ^]^ Copyright 2021, Wiley‐VCH.

M‐N‐C sites have been proposed as an important kind of active site in alcohol oxidation. Benefiting from atomic‐level heteroatom nitrogen and phosphorus doping, a Co_1_/P‐NC catalyst is reported to exhibit excellent activity and selectivity for selective oxidation of benzyl alcohols.^[^ ^345^ ^]^ A novel approach has been proposed to synthesize nitrogen‐doped graphene supported Co SAC (Co_1_@NG) with Co concentration of 4.1 wt.% for benzyl alcohol oxidation through the activation of peroxymonosulfate (PMS) under mild conditions (**Figure** **18**c).^[^ ^346^ ^]^ The graphene‐confined Co single atoms coordinate with N atoms and are highly active for activating PMS, superior to graphene oxide (GO), nitrogen‐doped graphene (NG), and Co NPs loaded on NG (Co NP@NG).

Atomically cobalt dispersed onto nitrogen‐doped graphene (Co‐NG) has been prepared by heat‐treating cobalt salts and graphene oxide in an ammonia atmosphere for selective alcohol oxidation.^[^ ^347^ ^]^ The Co‐NG catalyst reaches high benzyl alcohol conversion (94.8%) and excellent selectivity for benzaldehyde (97.5%) after reaction for 5 h with an average TOF of over 500 h^−1^. DFT calculations reveal that oxygen atoms are weakly adsorbed on the Co center and activated by the electron transfer from Co 3d orbitals to O_2_ 2p antibonding orbitals, forming superoxide species and abstracting the hydrogen of benzyl alcohol to yield benzaldehyde. A highly active Co‐N‐C SAC supported on graphite sheets is facilely synthesized for the selective aerobic oxidation of benzyl alcohol to benzaldehyde by a one‐pot method using inexpensive feedstocks of glucose, NH_4_Cl, and Co(NO_3_)_2_·6H_2_O.^[^ ^348^ ^]^ The atomical dispersion of Co atoms is confirmed by AC‐STEM and the Co‐N coordination is revealed by high‐resolution electron energy loss spectroscopy (EELS). After reaction for 8 h, the Co‐N—‐C SAC achieves 93.8% benzyl alcohol conversion and 97% benzaldehyde selectivity.

Single‐atom Fe catalysts based on nitrogen‐doped graphitic carbons are also synthesized for the selective oxidation of benzyl alcohol to aldehyde by high‐temperature pyrolysis.^[^ ^349^ ^]^ By regulating the amount of carbon nitride in the preparation process, the Fe_1_/N‐C catalyst has adjustable nitrogen contents, properly controlling the electron transfer from Fe single atoms to N atoms and then to the N‐doped carbon support. Fe single atoms are coordinated with N atoms and form FeN_4_ active identities, causing a decrease in the electron density of Fe, which facilitates the selective oxidation of benzyl alcohol to benzaldehyde.

The catalytic activities of Pd single atoms and clusters supported on CeO_2_ for benzyl alcohol oxidation are compared.^[^ ^350^ ^]^ Pd_1_/CeO_2_ achieves a high TOF of 6739 h^−1^ with a selectivity of almost 100 % for benzaldehyde, whereas the catalyst of Pd clusters (Pd_6_/CeO_2_) shows no activity. Theoretical calculations indicate that the interaction between hydroxy groups and CeO_2_ surface is prevented on Pd_6_/CeO_2_, which leads to its low activity, demonstrating that the isolated Pd atoms are the real active species.

The active sites in alcohol oxidation are studied by comparing three different Au species (single atoms, nanoclusters, and NPs of Au) supported on CeO_2_ nanorods (CeO_2_‐NR).^[^ ^351^ ^]^ Characterization results and DFT calculations suggest that the size of Au species and the concentration of oxygen vacancies (O_v_) significantly affect the activity of Au/CeO_2_ catalysts. In Au SAC, the much higher activity compared with catalysts of nanoclusters and NPs is attributed to the abundant [O‐O_v_‐Ce‐O‐Au] active sites, where the oxygen vacancies can promote the adsorption of benzyl alcohol and the splitting of the O‐H bond while Au^3+^/Au^+^ species tune the intermediates in the reaction.

Cinnamaldehyde can be synthesized in high selectivity through the selective oxidation of its corresponding alcohols^[^ ^352^ ^]^ to be used as pesticides^[^ ^353^ ^]^ and perfume or flavoring components.^[^ ^354^ ^]^ SACs have exhibited high activity towards this transformation. Mesoporous alumina‐based Pd catalysts are synthesized for the selective oxidation of cinnamyl alcohol under mild conditions. By reducing the loading of Pd species from 4.7 wt.% to 0.03 wt.%, the size of Pd species decreases and forms isolated Pd^II^ sites, which contributes to the high activity of 0.03 wt.% Pd_1_/meso‐Al_2_O_3_ achieving a TOF of 4400 h^−1^. Downsizing palladium species from metal clusters to isolated Pd^II ^sites supported on mesoporous alumina leads to a 30‐fold improvement in the activity per Pd atom. It's noteworthy that a high TOF of 4096 h^−1^ for the oxidation of benzyl alcohol with an over 99% selectivity is also obtained on the atomically dispersed Pd^II^ sites.^[^ ^24^ ^]^

Similarly, Al_2_O_3_‐supported Pd single atoms (Pd_1_/Al_2_O_3_ SAC) are reported to exhibit higher activity and selectivity than those of Pd NPs supported on Al_2_O_3_ (Pd/Al_2_O_3_) in cinnamyl alcohol oxidation.^[^ ^96^ ^]^ The Pd_1_/Al_2_O_3_ SAC reaches around 92% conversion of cinnamyl alcohol and high selectivity of 91% to cinnamaldehyde under 1 atm at 80 °C after an 8h reaction. By contrast, only 29% conversion with 89% selectivity is obtained on Pd/Al_2_O_3_. The excellent activity of Pd_1_/Al_2_O_3_ SAC is attributed to the unique electronic state of the isolated Pd atoms, which is revealed by XPS, XANES, and DRIFTs. An O_2_ activation mechanism is proposed that active oxygen species behaving like singlet oxygen are formed on Pd_1_/Al_2_O_3_ and then the partially dehydrogenated intermediates are oxidized to the target product.

As discussed above, with maximum amounts of uniform interfacial sites, SACs boost the selective oxidation of alcohols, showing higher activity and selectivity than that of metal NP catalysts (Table 2). The strategy of atomically dispersed metal‐support sites provides an inspiring approach for improving the catalytic performance of catalysts used in alcohol oxidation and other reactions.^[^ ^14^ ^]^

**Table 2:** Comparison of SACs and NP/nanocluster Catalysts for Selective Oxidation

Selective oxidation of methane to C1 Oxygenates on noble metals								
Catalyst	Gas	Oxidant	*T* [°C]	*P* [MP^a^]	Sel. [%]	Yield [mmol g_cat._^−1^ h^−1^]	TOF [h^−1^]	Ref
Rh_1_/ZrO_2_	95% CH_4_/He	H_2_O_2_	70	3	78.4	0.76	1.3	[315]
Rh/ZrO_2_	95% CH_4_/He	H_2_O_2_	70	3	55	/	/	
Rh_1_/CeO_2_‐NWs	CH_4_	H_2_O_2_	50	0.5	91	1232^a)^	127	[46]
Rh/CeO_2_‐NWs	CH_4_	H_2_O_2_	50	0.5	56.4	/	/	
Pd_1_/ZSM‐5	CH_4_	H_2_O_2_	95	3	96.2	7.69	8.3	[324]
Pd_1_/ZSM‐5 +2 wt% CuO	CH_4_	H_2_O_2_	95	3	86.4	7.88	/	
Au_1_/BP	CH_4_/O_2_ = 10/1	O_2_	90	3.3	>99%	≈0.057	5.6	[314]
Au NPs/BP	CH_4_/O_2_ = 10/1	O_2_	90	3.3	85	≈0.028	3.3	

Selective oxidation of methane to C1 Oxygenates on non‐noble metals								
Catalyst	Gas	Oxidant	*T* [°C]	*P* [MP^a^]	Sel. [%]	Yield [mmol g_cat._^−1^ h^−1^]	TOF [h^−1^]	Ref
Fe_1_N_4_/GN	89.9% CH_4_/N_2_	H_2_O_2_	25	2	94	0.47	0.23	[328]
0.03 wt% Fe_1_/ZSM‐5	90% CH_4_/N_2_	H_2_O_2_	80	3	94	421	84 200	[329]
2.6 wt% Fe/ZSM‐5	90% CH_4_/N_2_	H_2_O_2_	80	3	93	422	906	
7.8 wt% Fe/ZSM‐5	90% CH_4_/N_2_	H_2_O_2_	80	3	91	271	195	
Cu_1_/ZSM‐5	CH_4_	H_2_O_2_	50	3	99	9.6	157	[48]
Cu‐NP/ZSM‐5	CH_4_	H_2_O_2_	50	3	/	5	/	
1% Cr_1_/TiO_2_	CH_4_	H_2_O_2_	50	3	93	4.39	2.9	[332]
8% Cr/TiO_2_	CH_4_	H_2_O_2_	50	3	/	0.46	/	

Selective oxidation of benzyl alcohol to benzaldehyde								
Catalyst	Solvent	Oxidant	*T* [°C]	Conv. [%]	Sel. [%]	Yield [%]	TOF [h^−1^]	Ref
Co_1_/P‐NC	toluene	H_2_O_2_	110	99	99	98	/	[345]
Co NPs/NC	toluene	H_2_O_2_	110	10	/	/	/	
Co_1_@NG	acetonitrile/water (1:1)	PMS	50	90.6	93.4	84.7	/	[346]
Co—NP@NG	acetonitrile/water (1:1)	PMS	50	37.9	92.1	34.9	/	
Co_1_‐NG‐750	DMF	O_2_	130	94.8	97.5	92.4	544	[347]
Co_1_‐NC‐H‐800	*n*‐heptane	O_2_	90	93.8	97	91	37.0	[348]
Fe_1_/N_4.5_‐C	water	O_2_	100	92.7	96.4	89.4	3.4	[349]
Pd_1_/CeO_2_	/	O_2_	100	26.6	100	26.6	6739	[350]
Pd_6_/CeO_2_	/	O_2_	100	0	0	0	/	
Au_1_/CeO_2_‐NR	toluene	O_2_	100	89	94	84	193	[351]
Au‐NC/CeO_2_‐NR	toluene	O_2_	100	44	/	/	72	
Au‐NP/CeO_2_‐NR	toluene	O_2_	100	26	/	/	53	

Selective oxidation of cinnamyl alcohol to cinnamaldehyde								
Catalyst	Solvent	Oxidant	*T* [°C]	Conv. [%]	Sel. [%]	Yield [%]	TOF [h^−1^]	Ref
0.03 wt% Pd_1_/meso‐Al_2_O_3_	toluene	O_2_	60	/	92	/	822^b)^	[24]
1.03 wt% Pd/meso‐Al_2_O_3_	toluene	O_2_	60	/	91	/	50^b)^	
4.70 wt% Pd/meso‐Al_2_O_3_	toluene	O_2_	60	/	89	/	16^b)^	
Pd_1_/Al_2_O_3_	toluene	O_2_	80	92	91	84	775	[96]
Pd/Al_2_O_3_	toluene	O_2_	80	29	89	26	50	

^a)^ Yield (mmol_C1 oxygenates_ g_metal_^−1^ h^−1^)

^b)^ TON

## 5 Bridging Heterogeneous and Homogeneous Catalysis

According to the phases in which a catalyst and the reactant(s) exist, catalysis can be classified as homogeneous, heterogeneous, and biological.^[^ ^66^ ^]^ Compared to heterogeneous catalysts, homogeneous catalysts usually possess high activity and/or selectivity, due to the well‐designed structure and good solubility in the reaction medium.^[^ ^355^, ^356^, ^357^ ^]^ However, the difficult separation and recycling of homogeneous catalysts from a reaction mixture are the major drawbacks, which limit their industrial applications.^[^ ^16^, ^358^ ^]^ On the contrary, heterogeneous catalysts can be facilely separated from raw materials and products by attachment of metal clusters, NPs, or single atoms to an insoluble support, thus having good recovery and recyclability.^[^ ^66^ ^]^ SACs usually exhibit better catalytic activity and selectivity for a family of specific reaction systems than their NP and nanocluster counterparts owing to their unsaturated coordination environment and the homogeneity of active sites,^[^ ^34^, ^145^, ^148^ ^]^ and have been believed to be a bridge between heterogenous and homogenous catalysis.^[^ ^12^, ^16^ ^]^ Herein, enzyme‐like SACs, Suzuki reaction, and hydroformylation of olefins will be discussed as typical examples of SACs combining advantages of both heterogeneous and homogenous catalysis.

### 5.1 Enzyme‐like Catalysts

Natural enzymes participate widely in chemical and biological reactions and possess high efficiency and specificity.^[^ ^365^, ^366^ ^]^ Due to the expensive purification of natural enzymes and their instability in a harsh environment, nanomaterials with enzyme‐like properties as artificial enzymes have been extensively researched.^[^ ^367^, ^368^ ^]^ However, the diverse size and morphology of nanomaterials lead to the irregular distribution of active sites, resulting in much lower activity and selectivity compared to natural enzymes.^[^ ^369^ ^]^ Furthermore, the activity sites of nanozymes remain unclear, leading to the challenge of gaining insight into enzyme‐like activity mechanisms. SACs with well‐defined active sites can provide a potential pathway to improve the catalytic activity of nanozymes and offer good recyclability, serving as a bridge linking homogeneous catalysis and heterogeneous catalysis.^[^ ^362^, ^370^ ^]^

At present, carbon‐based SACs have raised great interest to mimic enzymes because of their favorable stability, flexibility with dopants, and good electrical conductivity.^[^ ^371^ ^]^ N‐Doped carbon materials including nanowires, nanotubes, nanosheets, and MOFs^[^ ^372^, ^373^ ^]^ have been used to prepare carbon‐supported SACs with uniform M‐N‐C sites to mimic natural metalloenzymes.^[^ ^374^ ^]^

Fe—N—C SAzymes are synthesized by dispersing single Fe atoms in carbon nanowire‐derived and nanotube‐derived materials. With atomically dispersed Fe atoms in polypyrrole (PPy) derived carbon nanowire, the SAzymes show high heme enzyme‐like property for H_2_O_2_ with an excellent specific activity (42.8 U mg^−1^).^[^ ^375^ ^]^ Owing to the large surface area of the carbon supports and the 100% utilization of isolated Fe‐N_x_ active sites, PPy‐derived carbon nanowire‐supported Fe‐N‐C SAzymes possess a similar structure with natural metalloproteases and optimized peroxidase‐like properties.^[^ ^376^, ^377^ ^]^ The TON of Fe‐N‐C SAzymes can reach 135 cycles in 5 min, which is 4500 times higher than classical Fe_3_O_4_ nanozyme.^[^ ^377^ ^]^ Moreover, the efficient SAzymes show the great potential of applying in immunoassay and sensor areas with better thermal and pH stable catalytic properties compared to natural enzymes.

**Figure 19:**
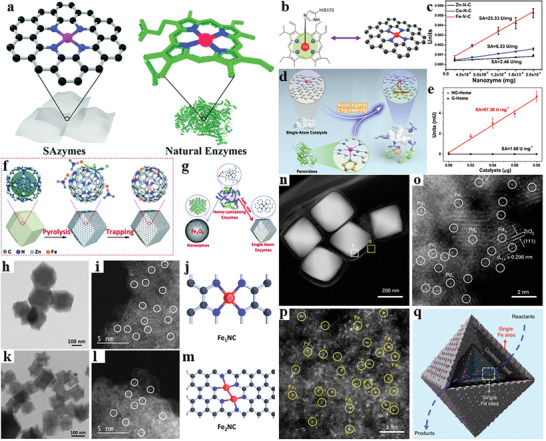
Enzyme‐like Catalysts. a) SAzymes mimicking natural enzymes. Reproduced with permission.^[^ ^359^ ^]^ Copyright 2021, Royal Society of Chemistry. b–e) SAzymes based on nitrogen‐doped carbon nanosheets. (b) Structures of natural peroxidase and Fe—N—C SACs. (c) Specific activities of M—N—C SAzymes (M = Fe, Co, Zn). Adapted with permission.^[^ ^360^ ^]^ Copyright 2020, American Chemical Society. (d) Schematic illustration of the axial‐ligand engineering. (e) Specific activities of FeN_5_ SAC (NG‐Heme) and FeN_4_ SAC (G‐Heme). Reproduced with permission.^[^ ^361^ ^]^ Copyright 2021, American Chemical Society. f–m) MOF‐based Fe SAzymes. (f) Schematic illustration of the synthesis of Fe SAEs. (g) Macrostructures and active sites of natural enzymes, nanozymes, and Fe SAEs. Reproduced with permission.^[^ ^362^ ^]^ Copyright 2019, Royal Society of Chemistry. (h) TEM images of Fe_1_NC nanozymes. (i) AC‐HADDF‐STEM images of Fe_1_NC nanozymes. (j) Active sites of Fe_1_NC nanozymes. (k) TEM images of Fe_2_NC nanozymes. (l) AC‐HADDF‐STEM images of Fe_2_NC nanozymes. (m) active sites of Fe_2_NC nanozymes. Reproduced with permission.^[^ ^363^ ^]^ Copyright 2021, Springer Nature. n–q) A yolk‐shell Pd_1_@Fe_1_ biomimetic composite. (n) HAADF‐STEM image of the yolk‐shell Pd_1_@Fe_1_. (o,p) Aberration‐corrected HAADF‐STEM images of the Pd_1_ (o) and Fe_1_ (p) sites in (n) (white and yellow squares, respectively). (q) Schematic sketch of the yolk‐shell nanostructure of Pd_1_@Fe_1_. Reproduced with permission.^[^ ^364^ ^]^ Copyright 2021, Springer Nature.

Attempts to construct and regulate single‐atom sites in SAzymes have also been made on nitrogen‐doped carbon nanosheets with abundant atomic fixation sites. Fe single atoms of high concentration (13.5 wt.%) anchored on ultrathin nitrogen‐doped carbon nanosheets exhibit excellent peroxidase‐like activities.^[^ ^360^ ^]^ As shown in **Figure** **19**c, the specific activity of Fe‐N‐C SAzymes (25.33 U mg^−1^) exceeds that of Zn‐N‐C SAzymes (2.46 U mg^−1^) and Co‐N‐C SAzymes (6.33 U mg^−1^). DFT calculations indicate that the difference in activity is remarkably correlated with charge distributions around the M_1_N_4_ sites (M = Fe, Co, Zn). This study provides guidelines to fabricate densely isolated M_1_N_4_ active sites and a perception of how M_1_N_4_ sites mimic natural peroxidase. Additionally, graphene‐supported SAzymes show high enzyme‐like activities through regulations of active sites. FeN_5_ SAzymes with an axial coordination ligand is prepared by assembling hemin on ultrathin nitrogen‐doped graphene (Figure 19d,e).^[^ ^361^ ^]^ The interaction between Fe‐N‐C sites and reaction intermediates is enhanced via the push effect, boosting the ability to activate H_2_O_2_ and achieving a vivid mimic of the active sites of peroxidase. With isolated active sites anchored on graphene layers, Fe‐N‐C SAzymes exhibit oxidase‐like properties by activating O_2_ into O_2_^−^· radicals and can be applied in biosensors.^[^ ^378^ ^]^

MOFs‐derived nitrogen‐doped carbon is also used as support materials to construct M‐N‐C sites. It is reported that Fe single atoms hosted by ZIF‐8 are prepared through a high‐temperature gas‐migration strategy (Figure 19f,g).^[^ ^362^ ^]^ Test experiments and characterization results confirm that the abundant and well‐defined porphyrin‐like FeN_4_ sites can interact strongly with oxygen species, exhibiting excellent peroxidase, oxidase, and catalase enzyme‐like activities. The calculated specific activity value of Fe single‐atom enzymes (SAEs) (6.75 U mg^−1^) is 40 times higher than that of Fe_3_O_4_ nanozymes (0.17 U mg^−1^). Another Fe‐N‐C single‐atom nanozyme exhibits impressive peroxidase‐like activity.^[^ ^379^ ^]^ The SAE is composed of uniform Fe‐N_x_ moieties that are atomically dispersed onto ZIF‐8, providing an almost comparable specific activity of 57.76 U mg^−1^ to natural horseradish peroxidase (HRP), which is far higher than that of typical Fe_3_O_4_ NPs, carbon NPs, and Au NPs. It is proposed that instead of the low utilization of atoms in a common nanozyme, the 100% atomic dispersion makes every single‐atom Fe combined with N an active site for the activation of H_2_O_2_, contributing to the greatly enhanced catalytic activity.

Moreover, atomically dispersed Fe‐Fe dual‐sites supported by MOF are reported to show higher oxidase‐like and peroxidase‐like activities than Fe_1_‐N‐C single atom enzymes.^[^ ^363^ ^]^ The XAFS results and theoretical calculations indicate that atomically dispersed Fe_2_N_6_ entities are formed in Fe_2_‐N‐C nanozymes while each Fe single atom is combined with four N atoms in Fe_1_‐N‐C nanozymes, as displayed in Figure 19j,m. Compared to the superoxo‐like configuration of O_2_ observed on the Fe_1_N_4_ sites, the peroxo‐like O_2_ absorption on Fe‐Fe dual‐sites could extend the O‐O bond length, which promotes the O‐O activation and boost the enzyme‐like activities of Fe_2_‐N‐C nanozymes.

It opens a possible new pathway to optimize SAzymes by doping phosphorus or boron in the FeN_x_ sites to further modulate the electronic structure of active entities. FeN_3_P‐centered SAzymes exhibit comparable peroxidase‐like activity and catalytic kinetics to natural enzymes by controlling the electronic structure of the single‐atom Fe active center through the precise coordination of phosphorus and nitrogen.^[^ ^380^ ^]^ Besides, theoretical calculations indicate that the intrinsic charge transfer in boron‐doped Fe‐N‐C single‐atom nanozymes can modulate the positive charge of the central Fe atom to reduce the energy barrier of the formation of hydroxyl radical and therefore boost the peroxidase‐like activity.^[^ ^381^ ^]^

The findings discussed above demonstrate the vivid simulation of natural enzyme structure through single‐atom strategy and provide great methods to accurately tune metal active centers for boosting nanozyme activities. In addition to directly mimicking the local structure of enzymatic active cites, it's of great significance to simulate the characteristics of enzyme catalysis and incorporate different reactions into one system like the more realistic biological system.

In natural systems, oxidation and reduction reactions catalyzed by different enzymes can take place within one cell‐like system with high efficiency and selectivity.^[^ ^382^ ^]^ A biomimetic composite called yolk‐shell Pd_1_@Fe_1_ is fabricated with atomically dispersed Fe_1_ sites in an N‐doped carbon shell and Pd_1_ sites in a MOF‐derived yolk (Figure 19n–q), which contains two compatible single‐atom systems.^[^ ^364^ ^]^ Their atomistic structures are verified by spherical aberration correction electron microscopy, XAFS analysis, and first‐principles. The yolk‐shell Pd_1_@Fe_1_ can catalyze nitroaromatic hydrogenation and alkene epoxidation reactions at the same time, motivating a cascade synthesis of amino alcohols. This catalyst shows the versatility that different single‐atom sites are incorporated into one system to continuously and facilely synthesize complex compounds.

In conclusion, SACs provide new approaches to finely regulate the structure to mimic the detailed structure of natural enzymes^[^ ^371^ ^]^ or the feature of enzyme catalysis in complex reaction systems,^[^ ^364^ ^]^ which is a promising direction to improve the efficiency of catalysts^[^ ^383^, ^384^ ^]^ and realize the combination of homo‐ and heterogeneous catalysis.^[^ ^385^ ^]^ Despite these breakthroughs, theoretical investigations into the design of efficient enzyme‐like SACs are still required for boosting their activities to reach natural enzymes’ high efficiency.

### 5.2 Suzuki Coupling Reaction

The palladium‐catalyzed Suzuki–Miyaura coupling reaction, which cross‐couples an organoboron reagent with an organohalide or sulfonates, is one of the most powerful methods for carbon‐carbon bond formation.^[^ ^386^, ^387^ ^]^ The low toxicity, wide compatibility with functional groups, stability in varied solvents, mild reaction conditions, and affordable reactants, have made Suzuki reaction extensively used in the preparation of pharmaceuticals^[^ ^388^, ^389^ ^]^ and total synthesis of natural products.^[^ ^390^ ^]^ Homogeneous systems use metal complexes to improve the kinetics but suffer from difficult separation of ligands and products.^[^ ^391^, ^392^ ^]^ Subsequently developed Pd NP catalysts lack sufficient activity due to less exposed active cites, and it is controversial whether the catalytic reaction mechanism is homogeneous or heterogeneous.^[^ ^393^, ^394^, ^395^, ^396^ ^]^ Through unremitting exploration, researchers find SACs provide opportunities for achieving good activity and recyclability simultaneously.

**Figure 20:**
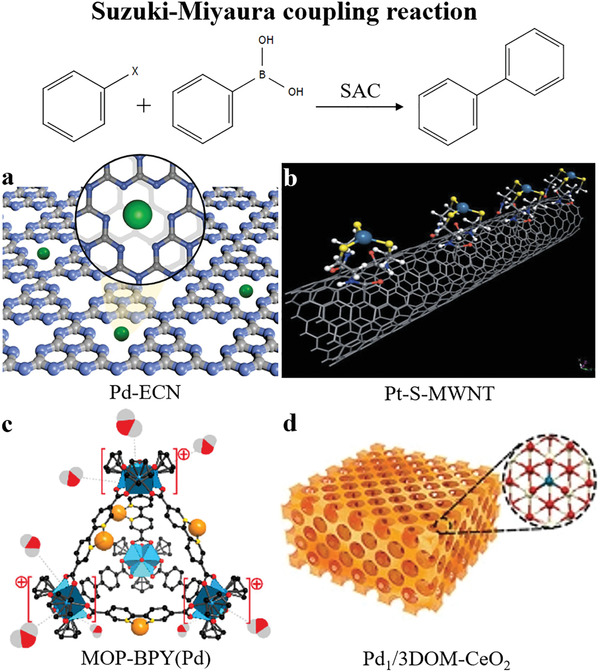
SACs for Suzuki–Miyaura coupling reaction. a) Schematic illustration of Pd‐ECN. Reproduced with permission.^[^ ^397^ ^]^ Copyright 2018, Springer Nature. b) Schematic illustration of Pt SAs surrounded by three sulfur atoms of thiol groups on the thiolated nanotube. Reproduced with permission.^[^ ^398^ ^]^ Copyright 2017, Elsevier. c) Schematic illustration for the composition and structure of MOP‐BPY(Pd). Reproduced with permission.^[^ ^399^ ^]^ Copyright 2020, Springer Nature. d) Schematic illustration of Pd_1_/3DOM‐CeO_2_ catalyst. Reproduced with permission.^[^ ^401^ ^]^ Copyright 2020, Wiley‐VCH.

Single palladium atoms anchored on exfoliated graphitic carbon nitride (Pd‐ECN) have been proposed as a stable heterogeneous catalyst with 90% selectivity and 56% purified yield of biphenyl exceeding the state‐of‐the‐art homogeneous catalysts for Suzuki coupling reaction.^[^ ^397^ ^]^ In the reaction process, Pd single atoms possess adaptive coordination with N sites of the ECN support, promoting the adsorption, stabilization, and activation of the substrates and intermediates. The ECN scaffold mimics the function of the ligands in homogeneous catalysts and its flexible lattice enables the variable coordination environment for Pd single atoms (**Figure** **20**a), which gives rise to high stability without metal leaching or aggregation.

Pt single atoms based on thiolated multi‐walled nanotubes have achieved a nearly complete conversion (more than 99% in 24h), surpassing that of homogeneous Pd or Pt catalysts (below 5% in 24h).^[^ ^398^ ^]^ Moreover, the catalyst is facile to be recollected by filtering and exhibits good recycling performance with 82% of initial yield after 12 cycles. Thiol groups introduced to carbon nanotubes prevent the Pt agglomeration and S atoms provide fast charge compensation for Pt to keep the electronic state close to zero (Figure 20b), which contributes to the excellent activity and recyclability.

Zr‐based metal‐organic polyhedra (MOPs) have been employed to confine Pd single atoms (MOP‐BPY(Pd)) to catalyze Suzuki–Miyaura coupling reaction in aqueous media (Figure 20c).^[^ ^399^ ^]^ The catalyst demonstrates over 90.0% yields for most substrates investigated, which outperforms Pd complex and MOF‐confined Pd atoms. Also, recollecting and recycling tests show no performance decline. It is discovered that each porous discrete cage of MOP‐BPY(Pd) contains 4.5 Pd atoms on average. MOP‐BPY(Pd) is highly dispersed in water and stable for three months, due to Zr‐based MOPs’ strong interactions with water,^[^ ^400^ ^]^ making it possible to catalyze efficient cross‐coupling reactions in aqueous media.

Pd SAC has also been constructed by anchoring Pd single atoms in 3D ordered porous ceria (Pd_1_/3DOM CeO_2_) via a pyrolysis method for Suzuki–Miyaura coupling reaction and displays good activity for various substrates under mild conditions (Figure 20d).^[^ ^401^ ^]^ It is another typical example of taking advantage of SMSIs in SACs to improve the efficiency and stability of heterogeneous catalyst systems. The CeO_2_ support provides a large surface area to anchor Pd single atoms, while Pd single atoms induce abundant oxygen vacancies and inhibit the grain growth of CeO_2_. No metal leaching and aggregation are observed after long‐term catalytic cycles, which attributes to the strong Pd‐O‐Ce bonds. Another palladium SAC which is based on bimetal oxides (Pd‐ZnO‐ZrO_2_) is synthesized through an in situ co‐precipitation method for Suzuki reaction under ambient conditions and shows high activity and tolerance of a broad scope of functional groups on substrates. Characterization indicates that each Pd atom is coordinated with two oxygen atoms, acting as the active sites. Moreover, the catalyst can be fabricated on a multi‐gram scale, exhibiting the potential to be developed for industrial applications.^[^ ^402^ ^]^

In conclusion, SACs have provided a promising approach to designing active and recyclable ligand‐free heterogeneous catalysts for the Suzuki–Miyaura reaction. By using various supporting materials to stabilize single atoms and regulate their electronic states through SMSIs, the advantages of both heterogeneous and homogenous catalysis are successfully combined.

### 5.3 Hydroformylation of Olefins

**Figure 21:**
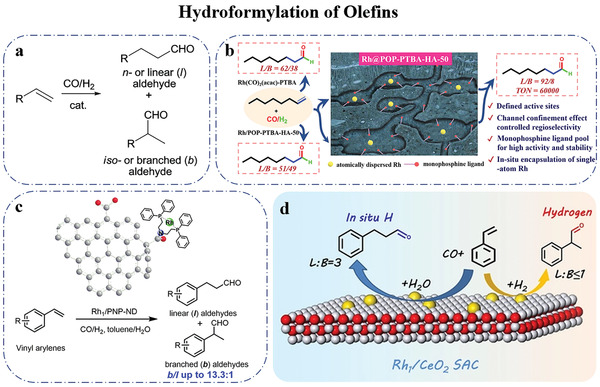
SACs catalyzing hydroformylation of olefins. a) Principle of Hydroformylation. Reproduced with permission.^[^ ^403^ ^]^ Copyright 2012, American Chemical Society. b) Isolated Rh atoms confined in monophosphine ligand pool for regioselective hydroformylation. Reproduced with permission.^[^ ^410^ ^]^ Copyright 2021, Elsevier. c) Rh_1_/PNP‐ND SAC for regioselective hydroformylation of vinyl arylenes. Reproduced with permission.^[^ ^409^ ^]^ Copyright 2021, Springer Nature. d) Hydroformylation of styrene to linear aldehydes over Rh_1_/CeO_2_ catalyst, which is coupled with the low‐temperature water‐gas shift reaction (LWGS) without the use of ligands. Reproduced with permission.^[^ ^411^ ^]^ Copyright 2020, Wiley‐VCH.

Hydroformylation of olefins that adds CO and H_2_ to olefins to form aldehydes is known as one of the most significant homogeneously catalyzed industrial processes (**Figure** **21**a).^[^ ^403^ ^]^ Homogeneous Rh complexes have been widely applied in this process for their superior activity and selectivity.^[^ ^404^, ^405^ ^]^ Because Rh complexes are hard to be separated from the products, heterogeneous catalysts of Rh NPs supported on carriers are developed but suffer from lower activities than the homogeneous counterparts.^[^ ^406^ ^]^ Therefore, as a new frontier of catalysis and a promising strategy for combining homogeneous and heterogeneous catalysis, SACs have exhibited their unique catalytic behaviors for the hydroformylation of olefins.

ZnO nanowires supported Rh single atoms (Rh_1_/ZnO SAC) demonstrate comparable or even superior activity to that of the classic homogenous Wilkinson's catalyst, RhCl(PPh_3_)_3_, for the hydroformylation of various olefins.^[^ ^355^ ^]^ The TON of the Rh_1_/ZnO SAC achieves around 40 000 while the TON of RhCl(PPh_3_)_3_ is about 19 000. XPS and XANES spectra show that single Rh atoms are in an almost metallic state, indicating that electrons transfer from Zn to Rh atoms, which stabilizes the catalyst and achieves good recyclability of 4 cycles without a significant decrease in the catalytic performance. The potential of Rh SACs supported on oxide for heterogeneous hydroformylation is further studied both theoretically and experimentally.^[^ ^407^ ^]^ Theoretical calculations indicate that Rh_1_/CeO_2_(111) is highly active and Rh_1_/ZnO is highly stable for hydroformylation. Then synthesized Rh_1_/CeO_2_ SAC exhibits comparable activity with that of HRh(PPh_3_)_3_CO catalyst and the Rh_1_/ZnO SAC exhibits excellent catalytic stability for the hydroformylation of olefin.

It has been reported that Rh_1_/CoO catalyst displays remarkable activity and selectivity for propene hydroformylation.^[^ ^408^ ^]^ Catalysts with rhodium loading rates of 0.2%, 1%, and 4.8% are prepared and it is found that metal clusters are formed in 1% Rh/CoO and 4.8% Rh/CoO. During the hydroformylation, the selectivity for butyraldehyde achieves 94.4% on 0.2% Rh_1_/CoO, superior to that of 1% Rh/CoO (68.7%) and 4.8% Rh/CoO (53.9%). Moreover, the TOF number of 0.2% Rh_1_/CoO (2065 h^−1^) is separately 1.4 and 5.2 times as that of 1% Rh/CoO and 4.8% Rh/CoO, and is comparable to homogeneous catalyst (5000 h^−1^).^[^ ^403^ ^]^ DFT calculations reveal that a structural reconstruction of Rh single atoms in Rh_1_/CoO occurs in the atmosphere containing H_2_ and CO and enhances the adsorption and activation of reactants. The reaction pathways are considered to be limited on Rh_1_/CoO, where only specific configurations are stable, thus effectively turning the selectivity of products.

Atomically dispersed Rh atoms in Rh_1_/PNP‐ND (phosphorus coordinated Rh single atoms supported on nanodiamond) as confirmed by HRTEM, ac‐HAADF‐STEM and CO‐DRIFTs results show excellent catalytic performance for the hydroformylation of arylethylenes with wide substrate generality.^[^ ^409^ ^]^ The Rh_1_/PNP‐ND catalyst reaches high conversion (> 99%) towards aldehyde and a high branched/linear aldehyde molar ratio of 13.3:1, which is commensurate with those of the homogeneous counterparts (Figure 21c). XAS and ^31^P solid‐state NMR reveal that Rh single atoms are stabilized by the bidentate chelation with two P atoms, contributing to the good stability of six cycles without activity and selectivity decreasing under a controlled conversion of 77%.

Porous monophosphine polymers confined to single Rh atoms possess a linear aldehyde selectivity of 92% for the hydroformylation of alkenes, which is much higher than that of the combination of Rh(CO)_2_(acac) and monophosphine ligand (PTBA).^[^ ^410^ ^]^ In the catalyst, single Rh atoms are stabilized by coordinating with the dispersive and abundant phosphine sites, forming precisely active sites comparable with homogeneous Rh complexes and contributing to the considerable catalytic activity without evident metal leaching (Figure 21b). The Rh SAC encapsulated in porous monophosphine polymers is easy to be recycled by centrifugation without obvious performance decline at the 3rd run.

An innovative idea of coupling the hydroformylation reaction with the water‐gas reaction to selectively produce linear aldehydes is proposed.^[^ ^411^ ^]^ Through this method, styrene and its derivatives are selectively transformed to linear aldehydes on an Rh SAC without the use of ligands (Figure 21d). The hydrogen formed in the low‐temperature water‐gas shift is considered to be crucial for the high ratio of linear/branched aldehyde. Under the same reaction coupling conditions, homogeneous catalysts of RhCl_3_ and RhCl(PPh_3_)_3_ exhibit similar high linear/branched aldehyde ratio with lower styrene conversion than that of Rh SAC, which is probably due to the lack of Rh‐CeO_2_ interfacial sites for the water‐gas reaction. This work provides a novel approach to bridging homogeneous and heterogeneous catalysis by applying SACs in processes that couple a classic homogeneous catalytic reaction with a heterogeneous catalyst.

On the whole, the great potential of applying SACs in organic transformations is proved experimentally and theoretically. SACs provide well‐defined active sites to obtain comparable activity and selectivity with that of traditional homogeneous catalysts while presenting good stability and recyclability like that of heterogeneous catalysts. The use of SACs in hydroformylation reaction provides new insights for bridging homogeneous and heterogeneous catalysis.

## 6 Conclusions and Perspectives

As what has been discussed and categorized above, the homogeneity of single‐atom active sites plays a significant role in tuning the activity and selectivity in various catalytic transformations, such as hydrogenation, oxidation, Suzuki coupling reaction, hydroformylation and so on. The uniform active sites composed of single‐atom sites facilitate the selective adsorption of specific functional groups and the activation of reactants. In the construction of enzyme‐like catalysts and various catalytic processes, SACs exhibit distinctive properties bridging homogeneous and heterogeneous catalysis. From current research on the evolution of the uniformity of single‐atom sites, it can be concluded that the stability of SACs is the premise for sustaining the high uniformity of single‐atom sites. Thus, efforts should be made to stabilize SACs via support defects engineering, spatial confinement, and doping strategies to sustain the original homogeneity. Moreover, by virtue of the uniform and well‐defined active sites of SACs, it is easier to identify the active sites and illustrate the structure‐activity relationship, further promoting exploration of the catalytic mechanism. From the aspect of this point, SACs provide a new platform for developing general knowledge to interpret the catalytic behaviors existing in homogeneous, heterogeneous, and enzyme catalysis so that better catalysts can be designed for practical applications crossing natural and man‐made fields.^[^ ^90^ ^]^ Nevertheless, the catalysts may experience a series of dynamic evolution in reaction processes, in which capturing key intermediates and unveiling the states and homogeneity (geometric, electronic structure, and local environments) of active sites is difficult. In this regard, more operando characterization techniques should be developed to obtain in situ information about homogeneity of active centers during the reaction processes.^[^ ^93^ ^]^ Tackling the fundamental questions of homogeneity and heterogeneity of active centers will continue relying on these advanced characterization techniques and theoretical calculations.

In terms of further improving the catalytic selectivity of active sites, we believe, in the premise of keeping the high homogeneity of active sites, precisely modulating the local coordination environments will be a promising direction. Based on existing studies of single‐atom active sites, it is considered that the active site of SACs should not be a sole metal atom but an active area centered by a single metal atom with nearby atoms (and/or vacancy, like oxygen vacancies)^[^ ^412^ ^]^ from the support surface, which is connected by MSIs. Hence, the coordination environments of the metal atoms by modulating CNs, introducing coordination heteroatoms, and selecting specific anchoring sites on substrates enable to significantly influence the adsorption behavior and binding energy of reactants and intermediates, which will regulate the distribution of products.^[^ ^413^, ^414^, ^415^, ^416^, ^417^ ^]^ It has been reported that for CO_2_ hydrogenation over the tailored Cu‐N_4_ and Cu‐N_3_ active sites, Cu‐N_3_ with 3‐coordinated N tends to promote CO production through the reverse water‐gas‐shift pathway and Cu‐N_4_ with 4‐coordinated N produces methanol with a high selectivity of 95.5% via the formate pathway.^[^ ^415^ ^]^ It has been reported that for CO_2_ electroreduction, single Sn atoms coordinating with O and N heteroatom (forming SnN_3_O_1_ configuration) enable to catalyze the formation of CO as the exclusive product while the single Sn atoms coordinate the N heteroatom (forming Sn‐N_4_ configuration) mainly produces HCOOH and H_2_.^[^ ^416^ ^]^ Due to the various topologies or defects of support, it has been reported that anchoring W single atoms on TiO_2_ atomic steps with O‐coordination (forming W^5+^‐O‐Ti^3+^ configuration) can selectively promote photocatalytic CO_2_ reduction to CH_4_ while W single atoms on Ti^3+^ (forming W^5+^‐Ti^3+^ configuration) sites mainly produce CO under the same reaction condition.^[^ ^413^ ^]^ Overall, it will be a promising way to improve the selectivity of target products by precisely tailoring local environments of single‐atom sites according to the property of reactants and supports.

Moreover, for the SACs with high‐density metal atoms, synergistic interactions between neighboring single metal atoms enable to efficiently promote the activation process of reactants on multiple atoms, which will be another strategy to improve the catalytic selectivity.^[^ ^363^, ^418^ ^]^ It has been reported that the neighboring single Pd atoms (with a single‐atom metal loading of 5.6%) display excellent activity and selectivity for carbon‐halogen bond splitting, which is superior to those of either sparsely dispersed Pd single atoms or Pd NPs.^[^ ^418^ ^]^ Also, synergistic effects of different kinds of single metal atoms have been proposed.^[^ ^199^, ^419^, ^420^ ^]^ For instance, Ir_1_Mo_1_/TiO_2_ dual‐SAC with two different single metal atoms exhibits much higher activity and selectivity (100% conversion with over 96% selectivity) than that of Ir_1_/TiO_2_ SACs (87% conversion with 38% selectivity) and Mo_1_/TiO_2_ (no activity) for the hydrogenation of 4‐nitrostyrene to 4‐vinylaniline. DFT calculations indicate that Ir single‐atom sites activate H_2_ molecules while Mo single‐atom sites adsorption of 4‐nitrostyrene, from which it can be speculated that different atomic active centers may potentially catalyze different reactions and further enhance total activity and selectivity. Therefore, designing dual‐single‐atom or multi‐SACs will be an efficient way to promote the catalytic selectivity of the complex reaction systems that require multiple active sites in real scenarios can be promoted by dual‐single‐atom or multi‐SACs utilizing the synergistic effect of neighboring atoms.^[^ ^421^ ^]^ Nevertheless, there is still limited knowledge about how the synergistic effect of multiple single atoms influences catalytic behaviors, which is worth continuously exploring.

Arguably, there is a long way to go before SACs are truly applied in industrial processes. First of all, the low density of single‐atom sites hinders the improvement of efficiency. If the loading concentration of single atoms is increased to obtain a high coverage of reactive sites, the extremely high efficiency of catalysts per volume or mass will be achieved with the maximum atomic utilization of SACs.^[^ ^422^, ^423^ ^]^ Second, how to fabricate SACs on a large scale is still challenging.^[^ ^424^ ^]^ Last but not least, as the reaction conditions can be diverse and complicated, it is of great significance to enhance the stability and sustain the original homogeneity of single‐atom active sites by acquiring deeper understanding to guide the rational design of applicable SACs.^[^ ^192^, ^425^ ^]^

In short, SACs have broad prospects with multiple challenges and encouraging opportunities. With joint efforts of researchers worldwide, it will be promising to better understand the concept and explore the dynamic evolution of the homogeneity of SACs, and develop appropriate strategies to sustain the homogeneity of SACs, which will benefit the development of economical, efficient, and environment‐friendly SACs toward a wider range of catalytic applications.

## Conflict of Interest

The authors declare no conflict of interest.
